# Supplementary material for: Hepatitis C care cascade among patients with and without tuberculosis: Nationwide observational cohort study in the country of Georgia, 2015–2020
Source: PLoS Med. 2023 May 4;20(5):e1004121. doi: 10.1371/journal.pmed.1004121 (PMC10194957; doi:10.1371/journal.pmed.1004121)
Supplement: S1 Analysis Plan — (DOCX) [file pmed.1004121.s003.docx]

**Section from dissertation proposal on hepatitis C care cascade among patients with and without TB**

# Hypothesis, rationale and preliminary data

We hypothesize that the proportion of HCV seropositive individuals who undergo HCV confirmatory testing, initiate and complete HCV treatment is lower among patients with TB compared to patients without TB. TB treatment is long, can last from 6 to 24 months (depending on whether the patient has drug susceptible or drug resistant disease) and treatment can be associated with drug-induced toxicity such as liver and kidney injury. Therefore, patients with TB might be more reluctant to start another long treatment course (8-24 weeks) due to previous negative treatment experience [1]. This issue has not been studied specifically among patients with both TB and HCV, but fatigue from diagnostic and treatment procedures as risk factor for treatment discontinuation has been described among patients with TB or those with TB/HIV coinfection [2-5]. This is even more likely because patients might not experience any symptoms associated with HCV for decades, so they might not feel the need to receive treatment and/or feel the urgency of seeking care for HCV [6].

Currently, patients with newly diagnosed TB in Georgia are routinely tested for HCV infection. According to the 2019 TB management guideline of Georgia, if HCV screening test is positive, blood samples are sent to NCDC for HCV confirmatory testing. However, there is no formal referral system in place linking patients with TB and confirmed HCV infection to HCV care. Unpublished data from NCDC shows that in 2019 of 213 HCV seropositive patients with TB, 86% underwent HCV confirmatory testing, but only 54% of those who had confirmed chronic HCV infection initiated HCV treatment in elimination program. However, these data do not provide details about timing of screening and confirmation or about any patients with TB in previous years. More in-depth analyses of the HCV care cascade and loss to follow-up among TB/HCV co-infected patients have not been conducted in Georgia nor elsewhere. Similar studies among HIV/TB co-infected patients suggest that linkage to specialized HIV care after positive test, as well as loss to follow-up during treatment is usually higher among co-infected patients compared to those with mono-infection [7-11]. Loss to follow-up is also common in the overall HCV care cascade in Georgia [12-15]. Among people with positive anti-HCV test results, only 80% proceed for confirmatory testing, and among those who were diagnosed with active HCV infection, only 80% start treatment. Overall, only 52% of persons with confirmed HCV infection have documented SVR, while the rest of the patients are lost to follow-up at different stages of care cascade.

# Design and study population

The study population for this aim will consist of patients diagnosed with TB who were also tested for HCV and had a positive HCV test anytime between 2 months before TB diagnosis date and end of TB treatment. We will identify a group of patients diagnosed with TB who are eligible for the HCV care cascade analysis by linking the TB surveillance database to the two HCV databases (described above). The main outcomes of interest are linkage to and retention in HCV care among patients with TB tested for HCV antibodies. Specifically, we will evaluate **four subsequent steps in the HCV care cascade**: 1. HCV confirmatory testing; 2. HCV treatment initiation; 3. HCV treatment completion; 4. HCV cure, defined as sustained virologic response (SVR) after 12 weeks of treatment completion. Patients will be classified as lost to follow-up after each step using the following criteria: 1. No confirmatory testing after >2 months after positive HCV screening test; 2. No treatment initiation after >4 months after HCV is confirmed; 3. Treatment initiated but not completed (assigned treatment completion status “incomplete” in the ElimC database); 4. >24 weeks is passed after treatment completion and SVR testing is not done. Care cascade in this group will be compared to findings from HCV care cascade among non-TB patients to evaluate if patients with TB are more likely to be lost to follow-up in HCV care compared to patients without TB.

# Statistical analysis

We will use two approaches to describe HCV care cascade among patients with TB and to compare it to patients without TB. First, we will calculate the proportion of patients with TB who tested positive on the anti-HCV test and subsequently reached each step in HCV care cascade, i.e. steps 1, 2, 3 and 4, described above. The comparison group will consist of individuals with anti-HCV positive test results without evidence of active TB. If a person is found to have TB treatment completed prior to first anti-HCV testing, they will be included in comparison non-TB care cascade. The proportion of patients reaching each subsequent step in care cascade will be compared between the two groups. In a second analysis, we will use survival analysis methods to explore if time to HCV confirmatory testing after positive screening test and time to HCV treatment initiation after positive confirmatory test is different between patients with and without TB. Patients will be censored at the end of follow-up or at the time of death. Kaplan-Meier curves will be created to graphically examine the differences between the two groups. Log rank test will be used to test for differences.

An additional analysis will be performed among patients with TB/HCV co-infection to identify demographic or TB-related factors associated with loss to follow-up. We will assess if the following patient characteristics are associated with higher risk of loss to follow-up in each step of care cascade: Age, sex, region of residence, employment, type of TB (drug-susceptible vs. drug-resistant), TB case definition (new vs retreatment case), and duration and outcome of TB treatment. Log-binomial regression will be used to calculate risk ratios and 95% confidence intervals.

**Table**. Changes in the analysis

| **Description of the change** | **Stage at which change occurred** | **Rationale of the change** |
| --- | --- | --- |
| Original design only mentioned that the study population would consist of patients diagnosed with TB who had a positive HCV antibody result. This was later changed to include the analysis of HCV antibody screening | After preliminary data analysis | It was suggested from some of the study team members that it is also necessary to understand the trends in HCV antibody testing among patients with TB. |
| Original definitions of LTFU included the following: No confirmatory testing after >2 months after positive HCV screening test; 2. No treatment initiation after >4 months after HCV is confirmed; We later decided to remove these time limits and count as LFU only those who did not proceed to the next stage of cascade at any time during the follow-up. | Before data analysis | Study team decided that original criteria put very strict time frame for LTFU, especially for patients with TB. Since majority of patients with TB get HCV screening at the beginning of their TB treatment, most of them have objective reasons for not getting confirmatory testing (before 2018, when patients had to get confirmatory testing themselves) or starting the treatment |
| Instead of censoring patients at the time of deaths, we used sudistribution hazards model that takes into account the competing risks. Additionally, instead of Kaplan-Meier curves and Log rank test, we used cumulative incidence curves and Gray's test for equality of cumulative incidence functions | After preliminary data analysis | Preliminary data analysis showed that nontrivial proportion of patients died during the follow-up. Therefore, we used the tests that are more recommended for the competing risks analysis. |
| Conducted null hypothesis significance testing and calculated the p-values for several analyses | Peer review | Requested by the academic editor |

**References:**

1. Aibana O, Dauria E, Kiriazova T, Makarenko O, Bachmaha M, Rybak N, et al. Patients’ perspectives of tuberculosis treatment challenges and barriers to treatment adherence in Ukraine: a qualitative study. BMJ Open. 2020;10(1). doi: 10.1136/bmjopen-2019-032027. PubMed PMID: 32014870.

2. Skinner D, Claassens M. It’s complicated: why do tuberculosis patients not initiate or stay adherent to treatment? A qualitative study from South Africa. BMC infectious diseases. 162016.

3. O’Donnell MR, Padayatchi N, Daftary A, Orrell C, Dooley KE, Amico KR, et al. Antiretroviral switching and bedaquiline treatment of drug-resistant tuberculosis HIV co-infection. Lancet HIV. 2019;6(3):e201-4. doi: 10.1016/s2352-3018(19)30035-9. PubMed PMID: 30846058; PubMed Central PMCID: PMC7155388.

4. O’Donnell MR, Daftary A, Frick M, Hirsch-Moverman Y, Amico KR, Senthilingam M, et al. Re-inventing adherence: toward a patient-centered model of care for drug-resistant tuberculosis and HIV. Int J Tuberc Lung Dis. 2016;20(4):430-4. doi: 10.5588/ijtld.15.0360. PubMed PMID: 26970149; PubMed Central PMCID: PMC4863995.

5. Furin J, Isaakidis P, Reid AJ, Kielmann K. 'I'm fed up': experiences of prior anti-tuberculosis treatment in patients with drug-resistant tuberculosis and HIV. Int J Tuberc Lung Dis. 2014;18(12):1479-84. Epub 2014/12/18. doi: 10.5588/ijtld.14.0277. PubMed PMID: 25517815.

6. Westbrook RH, Dusheiko G. Natural history of hepatitis C. J Hepatol. 2014;61(1 Suppl):S58-68. Epub 2014/12/03. doi: 10.1016/j.jhep.2014.07.012. PubMed PMID: 25443346.

7. Gezae KE, Abebe HT, Gebretsadik LG. Incidence and predictors of LTFU among adults with TB/HIV co-infection in two governmental hospitals, Mekelle, Ethiopia, 2009-2016: survival model approach. BMC infectious diseases. 2019;19(1):107. Epub 2019/02/06. doi: 10.1186/s12879-019-3756-2. PubMed PMID: 30717705; PubMed Central PMCID: PMCPMC6360725.

8. Bassett IV, Chetty S, Wang B, Mazibuko M, Giddy J, Lu Z, et al. Loss to follow-up and mortality among HIV-infected people co-infected with TB at ART initiation in Durban, South Africa. J Acquir Immune Defic Syndr. 2012;59(1):25-30. Epub 2011/10/27. doi: 10.1097/QAI.0b013e31823d3aba. PubMed PMID: 22027877; PubMed Central PMCID: PMCPMC3237807.

9. Pablos-Mendez A, Knirsch CA, Barr RG, Lerner BH, Frieden TR. Nonadherence in tuberculosis treatment: predictors and consequences in New York City. The American journal of medicine. 1997;102(2):164-70. PubMed PMID: 9217566.

10. Voss De Lima Y, Evans D, Page-Shipp L, Barnard A, Sanne I, Menezes CN, et al. Linkage to Care and Treatment for TB and HIV among People Newly Diagnosed with TB or HIV-Associated TB at a Large, Inner City South African Hospital. PloS one. 2013;8(1). doi: 10.1371/journal.pone.0049140. PubMed PMID: 23341869.

11. Maraba N, Chihota V, McCarthy K, Churchyard GJ, Grant AD. Linkage to care among adults being investigated for tuberculosis in South Africa: pilot study of a case manager intervention. BMJ Open. 2018;8(5). doi: 10.1136/bmjopen-2017-021111. PubMed PMID: 29794100.

12. Nasrullah M, Sergeenko D, Gvinjilia L, Gamkrelidze A, Tsertsvadze T, Butsashvili M, et al. The Role of Screening and Treatment in National Progress Toward Hepatitis C Elimination - Georgia, 2015-2016. MMWR Morb Mortal Wkly Rep. 2017;66(29):773-6. Epub 2017/07/28. doi: 10.15585/mmwr.mm6629a2. PubMed PMID: 28749925; PubMed Central PMCID: PMCPMC5657814.

13. Gvinjilia L, Nasrullah M, Sergeenko D, Tsertsvadze T, Kamkamidze G, Butsashvili M, et al. National Progress Toward Hepatitis C Elimination - Georgia, 2015-2016. MMWR Morb Mortal Wkly Rep. 2016;65(41):1132-5. Epub 2016/10/21. doi: 10.15585/mmwr.mm6541a2. PubMed PMID: 27764081.

14. Averhoff F, Lazarus JV, Sergeenko D, Colombo M, Gamkrelidze A, Tsertsvadze T, et al. Excellence in Viral Hepatitis Elimination - Lessons from Georgia. J Hepatol. 2019. Epub 2019/07/30. doi: 10.1016/j.jhep.2019.06.026. PubMed PMID: 31356831.

15. Averhoff F, Shadaker S, Gamkrelidze A, Kuchuloria T, Gvinjilia L, Getia V, et al. Progress and Challenges in a Pioneering Hepatitis C Elimination Program in the Country of Georgia, 2015-2018. J Hepatol. 2019. Epub 2019/12/08. doi: 10.1016/j.jhep.2019.11.019. PubMed PMID: 31811882.
