# Supplementary material for: Hepatitis C care cascade among patients with and without tuberculosis: Nationwide observational cohort study in the country of Georgia, 2015–2020
Source: PLoS Med. 2023 May 4;20(5):e1004121. doi: 10.1371/journal.pmed.1004121 (PMC10194957; doi:10.1371/journal.pmed.1004121)
Supplement: S2 Fig — (DOCX) [file pmed.1004121.s005.docx]

**S2 Fig**. Directed acyclic graph depicting the causal relations between exposure of interest (MDR TB), outcome of interest (loss to follow-up) and other covariates.


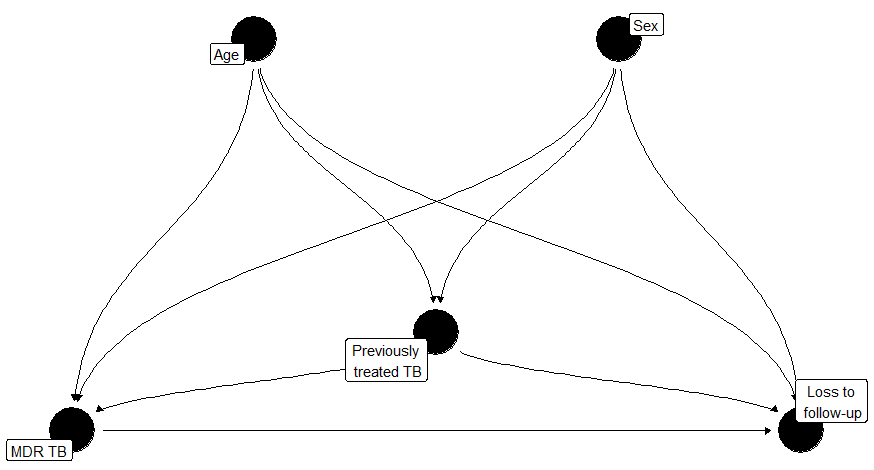


Abbreviations: TB, tuberculosis; MDR, multidrug-resistant.
