# Supplementary material for: Hepatitis C care cascade among patients with and without tuberculosis: Nationwide observational cohort study in the country of Georgia, 2015–2020
Source: PLoS Med. 2023 May 4;20(5):e1004121. doi: 10.1371/journal.pmed.1004121 (PMC10194957; doi:10.1371/journal.pmed.1004121)
Supplement: S6 Fig — Note: Red lines represent the percent change between 2 consecutive steps in the care cascade, i.e., adjacent bars of the charts. HCV, hepatitis C virus; SVR, sustained virologic response; TB, tuberculosis; Tx, treatment. (DOCX) [file pmed.1004121.s009.docx]

**S6 Fig**. Hepatitis C care cascade among HCV seropositive patients with TB, stratified by their drug-resistance status
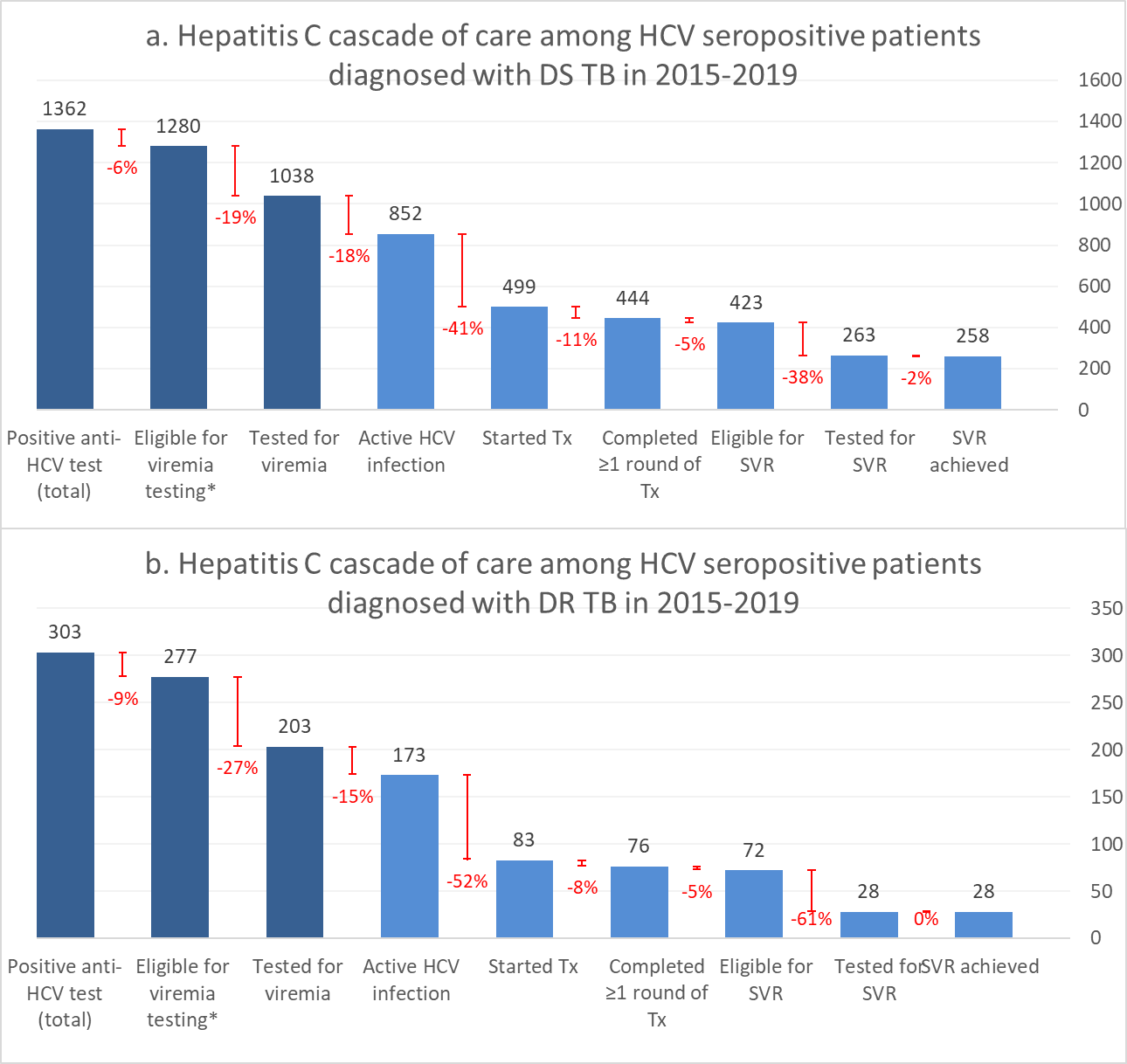


* No death before viremia testing

Abbreviations: TB, tuberculosis; HCV, hepatitis C virus; Tx, treatment; SVR, sustained virologic response;

Note: Red lines represent the percent change between two consecutive steps in the care cascade, i.e. adjacent bars of the charts.
