# Supplementary material for: Hepatitis C care cascade among patients with and without tuberculosis: Nationwide observational cohort study in the country of Georgia, 2015–2020
Source: PLoS Med. 2023 May 4;20(5):e1004121. doi: 10.1371/journal.pmed.1004121 (PMC10194957; doi:10.1371/journal.pmed.1004121)
Supplement: S7 Fig — HCV, hepatitis C virus; SVR, sustained virologic response; TB, tuberculosis; Tx, treatment. (DOCX) [file pmed.1004121.s010.docx]

**S7 Fig**. Comparison of hepatitis C care cascade among patients with and without TB in Georgia, 2015-2020.


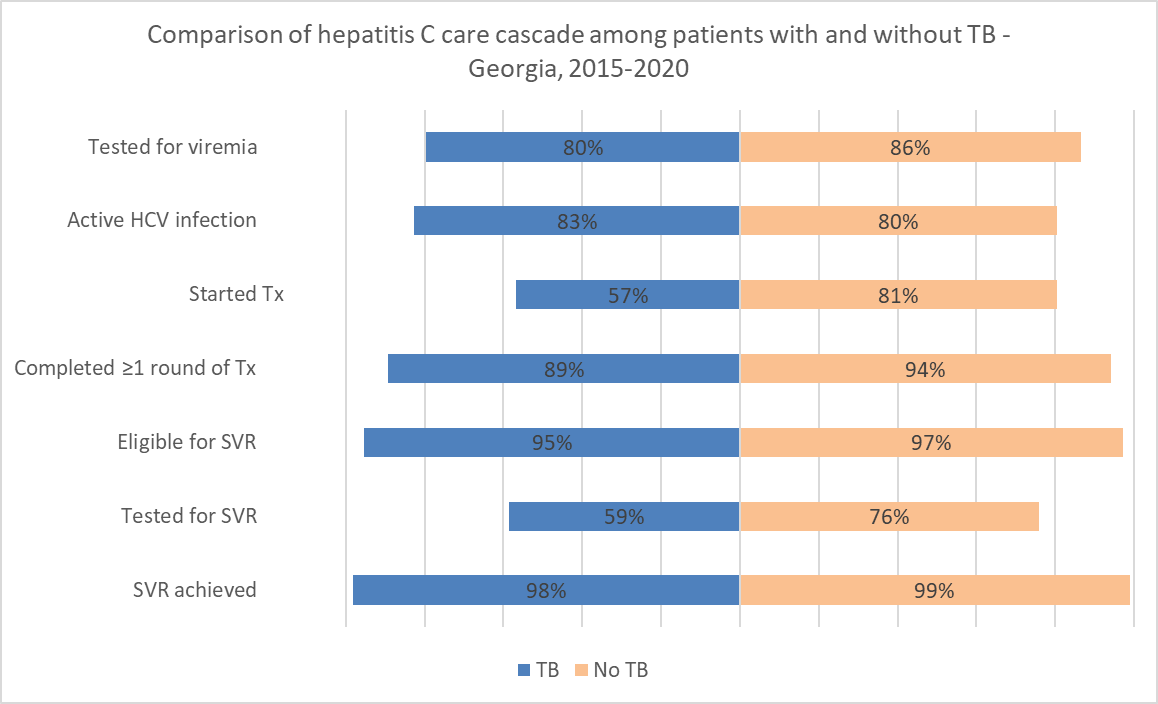


Abbreviations: TB, tuberculosis; HCV, hepatitis C virus; Tx, treatment; SVR, sustained virologic response;
