# Supplementary material for: Hepatitis C care cascade among patients with and without tuberculosis: Nationwide observational cohort study in the country of Georgia, 2015–2020
Source: PLoS Med. 2023 May 4;20(5):e1004121. doi: 10.1371/journal.pmed.1004121 (PMC10194957; doi:10.1371/journal.pmed.1004121)
Supplement: S8 Fig — HCV, hepatitis C virus; TB, tuberculosis. (DOCX) [file pmed.1004121.s011.docx]

**S8 Fig**. Kaplan-Meier curves of time from positive antibody test to viremia testing, with 95% confidence bands


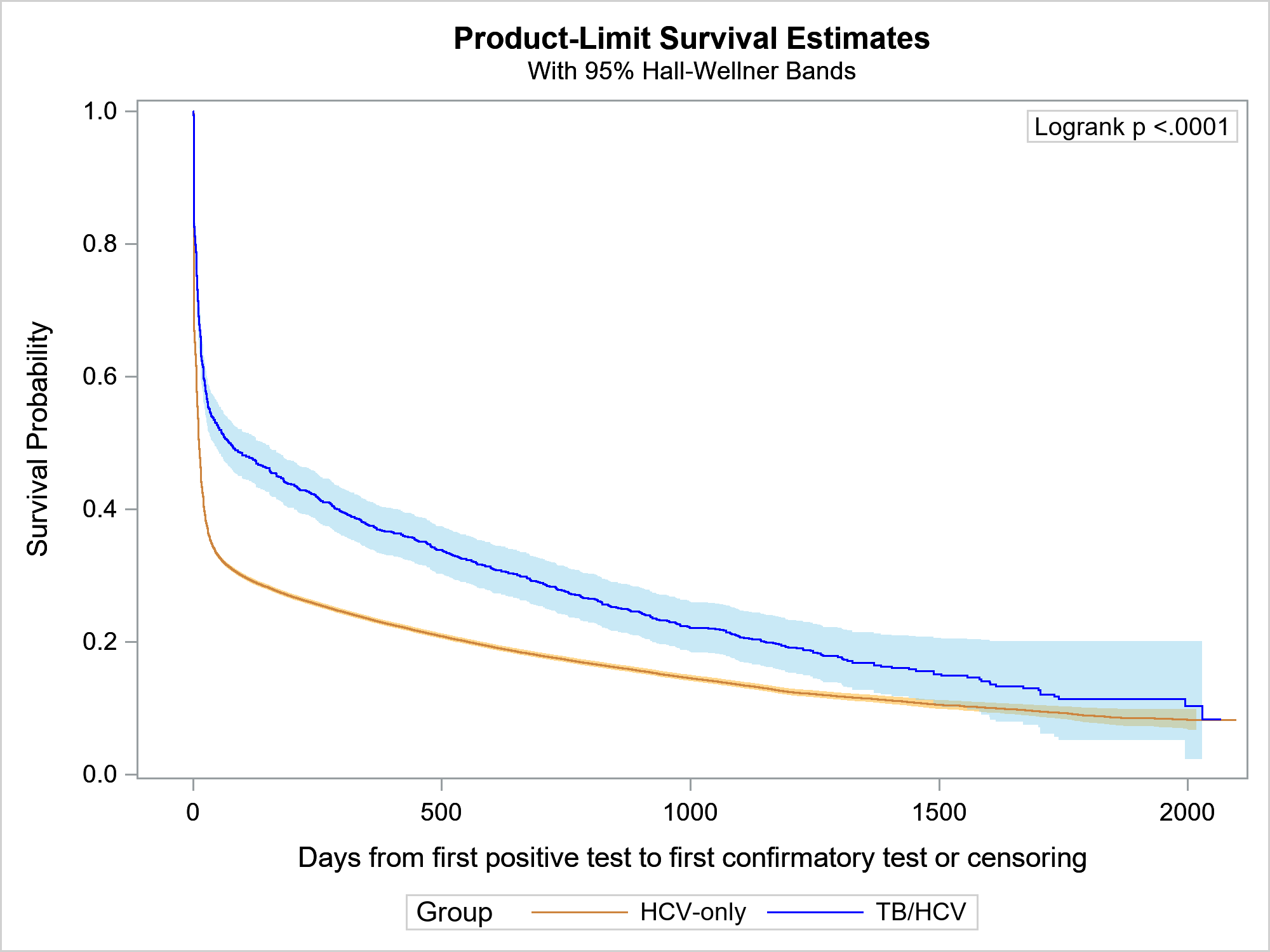


Abbreviations: TB, tuberculosis; HCV, hepatitis C virus;
