# Supplementary material for: Hepatitis C care cascade among patients with and without tuberculosis: Nationwide observational cohort study in the country of Georgia, 2015–2020
Source: PLoS Med. 2023 May 4;20(5):e1004121. doi: 10.1371/journal.pmed.1004121 (PMC10194957; doi:10.1371/journal.pmed.1004121)
Supplement: S1 Table — HCV, hepatitis C virus; SVR, sustained virologic response; TB, tuberculosis; Tx, treatment. (DOCX) [file pmed.1004121.s013.docx]

**S1 Table**. Comparison of proportions at each step of care cascade between patients with and without TB, Georgia, 2015-2020

| **Care cascade step** | **Total** |  | **TB** | | **No TB** | |  |
| --- | --- | --- | --- | --- | --- | --- | --- |
|  | **N** | **%** | **n** | **% (from previous step)** | **n** | **% (from previous step)** | **chi-square p-value** |
| Positive anti-HCV test (total) | 132502 | 7% | 1665 | 18% | 130837 | 7% |  |
| Eligible for viremia testing* | 128552 | 97% | 1557 | 94% | 126995 | 97% | <0.001 |
| Tested for viremia | 111001 | 86% | 1241 | 80% | 109760 | 86% | <0.001 |
| Active HCV infection | 89353 | 80% | 1025 | 83% | 88328 | 80% | 0.061 |
| Started Tx | 71740 | 80% | 582 | 57% | 71158 | 81% | <0.001 |
| Completed ≥1 round of Tx | 67551 | 94% | 520 | 89% | 67031 | 94% | <0.001 |
| Eligible for SVR | 65620 | 97% | 496 | 95% | 65124 | 97% | 0.016 |
| Tested for SVR | 49657 | 76% | 291 | 59% | 49366 | 76% | p<0.001 |
| SVR achieved | 49097 | 99% | 286 | 98% | 48811 | 99% | P=0.339 |

Abbreviations: TB, tuberculosis; HCV, hepatitis C virus; Tx, treatment; SVR, sustained virologic response;

* No death before viremia testing
