# Supplementary material for: Hepatitis C care cascade among patients with and without tuberculosis: Nationwide observational cohort study in the country of Georgia, 2015–2020
Source: PLoS Med. 2023 May 4;20(5):e1004121. doi: 10.1371/journal.pmed.1004121 (PMC10194957; doi:10.1371/journal.pmed.1004121)
Supplement: Attachment — Submitted filename: PMEDICINE-D-22-03278_R1_reviewer.pdf [file pmed.1004121.s014.pdf]

PLOS Medicine

Hepatitis C care cascade among patients with and without tuberculosis: findings from nationwide programs in the country of Georgia, 2015-2020

--Manuscript Draft--

**Manuscript Number:**	PMEDICINE-D-22-03278R1
**Full Title:**	Hepatitis C care cascade among patients with and without tuberculosis: findings from nationwide programs in the country of Georgia, 2015-2020
**Short Title:**	Hepatitis C care cascade among patients with and without TB
**Article Type:**	Research Article
**Abstract:**	Background<br><br>The Eastern European country of Georgia initiated a nationwide hepatitis C virus (HCV) elimination program in 2015 to address a high burden of infection. Screening for HCV infection through antibody testing was integrated into multiple existing programs, including the National Tuberculosis Program (NTP). We sought to evaluate loss to follow-up (LTFU) from the hepatitis C care cascade among persons diagnosed with active tuberculosis (TB) disease.<br><br>Methods<br><br>Using national ID numbers, we merged databases of the HCV elimination program, NTP, and national death registry from January 1, 2015, to September 30, 2020. We estimated the proportion of patients with and without TB who were LTFU at each step of the HCV care cascade and explored temporal changes.<br><br>Results<br><br>Among 11,985 patients with active TB, 9,065 (76%) were tested for HCV antibodies, and 1,665 (18%) had a positive result; LTFU from hepatitis C care was common, with 20% of patients with a positive antibody test not undergoing viremia testing, and 43% of patients with viremia not starting treatment for hepatitis C. Overall, among persons with confirmed viremic HCV infection, only 28% of patients with TB had a documented cure from HCV infection, compared to 55% among patients without TB. LTFU after positive antibody testing substantially decreased in the last three years, from 32% among patients diagnosed with TB in 2017 to 12% among those diagnosed in 2019.<br><br>Conclusions<br><br>LTFU from hepatitis C care after a positive antibody or viremia test was high and more common among patients with TB than in those without TB. Better integration of TB and hepatitis C care systems can potentially reduce LTFU and improve patient outcomes. Existing large-scale programs for both TB and hepatitis C in Georgia create a unique opportunity for such integration to contribute to hepatitis C elimination efforts.
**Additional Information:**	
**Question**	**Response**
**Financial Disclosure**<br><br>Enter a financial disclosure statement that describes the sources of funding for the work included in this submission. Review the submission guidelines for detailed requirements. View published research articles from *PLOS Medicine* for specific examples.	This work was supported in part from grants from the National Institutes of Health (NIH) Fogarty International Center (D43TW007124; “Emory-Georgia TB Research 12 Training Program”) to DB and from NIH/National Institutue of Allergy and Infectious Diseases (K24AI114444; the Emory/Georgia TB Research Advancement Center, P30AI168386; the Emory University Center for AIDS Research, P30AI050409; and TB Research Unit-ASTRa, U19AI111211) to NRG.

This statement is required for submission and **will appear in the published article** if the submission is accepted. Please make sure it is accurate.<br><br>**Unfunded studies**<br>Enter: *The author(s) received no specific funding for this work.*<br><br>**Funded studies**<br>Enter a statement with the following details:<br>• Initials of the authors who received each award<br>• Grant numbers awarded to each author<br>• The full name of each funder<br>• URL of each funder website<br>• Did the sponsors or funders play any role in the study design, data collection and analysis, decision to publish, or preparation of the manuscript?<br>• **NO** - Include this sentence at the end of your statement: *The funders had no role in study design, data collection and analysis, decision to publish, or preparation of the manuscript.*<br>• **YES** - Specify the role(s) played.<br><br>* typeset	
**Competing Interests**<br><br>Use the instructions below to enter a competing interest statement for this submission. On behalf of all authors, disclose any competing interests that could be perceived to bias this work—acknowledging all financial support and any other relevant financial or non-financial competing interests.<br><br>This statement **will appear in the published article** if the submission is accepted. Please make sure it is accurate. View published research articles from *PLOS Medicine* for specific examples.	The authors have declared that no competing interests exist.

**NO authors have competing interests**<br><br>Enter: *The authors have declared that no competing interests exist.*<br><br><br>**Authors with competing interests**<br>Enter competing interest details beginning with this statement:<br><br>*I have read the journal's policy and the authors of this manuscript have the following competing interests: [insert competing interests here]*<br><br><br><br>* typeset<br><br><br>This statement is required for submission and **will appear in the published article** if the submission is accepted. Please make sure it is accurate and that any funding sources listed in your Funding Information later in the submission form are also declared in your Financial Disclosure statement.	
**Ethics Statement**<br><br><br>You must include an Ethics Statement in the Methods section of your manuscript if your study involved:<br>• Human participants<br>• Human specimens or tissue<br>• Vertebrate animals or cephalopods<br>• Vertebrate embryos or tissues<br>• Field research<br><br>**Not including a statement when required will delay the review process.**<br><br><br>General guidance is provided below. Please consult the guidelines on human subjects research and animal research for detailed instructions.	I confirm that I have read and understood PLOS' requirements for an Ethics Statement.

Powered by Editorial Manager® and ProduXion Manager® from Aries Systems Corporation

**Format for specific study types**<br><br>**Human Subject Research (involving human participants and/or tissue)**<br>• Give the name of the institutional review board or ethics committee that approved the study<br>• Include the approval number and/or a statement indicating approval of this research<br>• Indicate the form of consent obtained (written/oral) or the reason that consent was not obtained (e.g. the data were analyzed anonymously)<br><br>**Animal Research (involving vertebrate animals, embryos or tissues)**<br>• Provide the name of the Institutional Animal Care and Use Committee (IACUC) or other relevant ethics board that reviewed the study protocol, and indicate whether they approved this research or granted a formal waiver of ethical approval<br>• Include an approval number if one was obtained<br>• If the study involved *non-human primates*, add *additional details* about animal welfare and steps taken to ameliorate suffering<br><br>**Field Research**<br><br>Include the following details if this study involves the collection of plant, animal, or other materials from a natural setting:<br>• Field permit number<br>• Name of the institution or relevant body that granted permission<br><br>Please check the box to confirm your understanding of this policy, then read and agree to one of the statements.	
Please respond:<br>as follow-up to "**Ethics Statement**<br><br>You must include an Ethics Statement in	My study requires an ethics statement. I confirm I have included my statement in the Methods section of my uploaded manuscript file.

the Methods section of your manuscript if your study involved:<br>• Human participants<br>• Human specimens or tissue<br>• Vertebrate animals or cephalopods<br>• Vertebrate embryos or tissues<br>• Field research<br><br>**Not including a statement when required will delay the review process.**<br><br>General guidance is provided below. Please consult the guidelines on human subjects research and animal research for detailed instructions.	

**Format for specific study types**<br><br>**Human Subject Research (involving human participants and/or tissue)**<br>• Give the name of the institutional review board or ethics committee that approved the study<br>• Include the approval number and/or a statement indicating approval of this research<br>• Indicate the form of consent obtained (written/oral) or the reason that consent was not obtained (e.g. the data were analyzed anonymously)<br><br>**Animal Research (involving vertebrate animals, embryos or tissues)**<br>• Provide the name of the Institutional Animal Care and Use Committee (IACUC) or other relevant ethics board that reviewed the study protocol, and indicate whether they approved this research or granted a formal waiver of ethical approval<br>• Include an approval number if one was obtained<br>• If the study involved *non-human primates*, add *additional details* about animal welfare and steps taken to ameliorate suffering<br><br>**Field Research**<br><br>Include the following details if this study involves the collection of plant, animal, or other materials from a natural setting:<br>• Field permit number<br>• Name of the institution or relevant body that granted permission<br><br>Please check the box to confirm your understanding of this policy, then read and agree to one of the statements. "	
**Data Availability**<br><br>Authors are required to make all data underlying the findings described fully available, without restriction, and from the time of publication. PLOS allows rare exceptions to address legal and ethical concerns. See the PLOS Data Policy and	No - some restrictions will apply

FAQ for detailed information.<br><br>A Data Availability Statement describing where the data can be found is required at submission. Your answers to this question constitute the Data Availability Statement and **will be published in the article**, if accepted.<br><br>**Important:** Stating 'data available on request from the author' is not sufficient. If your data are only available upon request, select 'No' for the first question and explain your exceptional situation in the text box.<br><br>Do the authors confirm that all data underlying the findings described in their manuscript are fully available without restriction?	
**Describe where the data may be found in full sentences. If you are copying our sample text, replace any instances of XXX with the appropriate details.**<br><br>• If the data are **held or will be held in a public repository**, include URLs, accession numbers or DOIs. If this information will only be available after acceptance, indicate this by ticking the box below. For example: *All XXX files are available from the XXX database (accession number(s) XXX, XXX.*).<br>• If the data are all contained **within the manuscript and/or Supporting Information files**, enter the following: *All relevant data are within the manuscript and its Supporting Information files.*<br>• If neither of these applies but you are able to provide **details of access elsewhere**, with or without limitations, please do so. For example:<br><br>*Data cannot be shared publicly because of [XXX]. Data are available from the XXX Institutional Data Access / Ethics Committee (contact via XXX) for*	The data used in this analysis are not owned by the authors and therefore cannot be shared publicly. The requests for the data sharing should be sent to the National Center for Disease Control and Public Health of Georgia via email: pr.ncdc@ncdc.ge, and to the National Center for Tuberculosis and Lung Disease via email: tbcenter@tbgeo.ge. We confirm that others researchers who meet the criteria for access to confidential data will be able to access these data in the same manner as the authors. The authors did not have any special access privileges that others would not have.

researchers who meet the criteria for access to confidential data.<br><br>*The data underlying the results presented in the study are available from (include the name of the third party and contact information or URL).*<br>• This text is appropriate if the data are owned by a third party and authors do not have permission to share the data.<br><br>* typeset	
Additional data availability information:	

Powered by Editorial Manager® and ProduXion Manager® from Aries Systems Corporation

Full manuscript

Click here to access/download;Manuscript;TB_HCV_care_cascade_manuscri

Title: Hepatitis C care cascade among patients with and without tuberculosis: findings from nationwide programs in the country of Georgia, 2015-2020

Authors: Davit Baliashvili,[1] Henry M. Blumberg,[1,2,3] Neel R. Gandhi,[1,2,3] Francisco Averhoff,[4] David Benkeser,[5] Shaun Shadaker,[6] Lia Gvinjilia,[7] Aleksandre Turdziladze,[8] Nestani Tukvadze,[9] Mamuka Chincharauli,[9] Maia Butsashvili,[10] Lali Sharvadze,[11,12] Tengiz Tsertsvadze,[13] Jaba Zarkua,[14] Russell R. Kempker[3]

Affiliations:

1. Department of Epidemiology, Emory University Rollins School of Public Health, Atlanta, GA, USA;

2. Department of Global Health, Emory University Rollins School of Public Health, Atlanta, GA, USA;

3. Division of Infectious Diseases, Department of Medicine, Emory University School of Medicine, Atlanta, GA, USA,

4. Department of Family and Preventive Medicine, Emory University School of Medicine;

5. Department of Biostatistics and Bioinformatics, Emory University Rollins School of Public Health, Atlanta, GA, USA;

6. Division of Viral Hepatitis, Centers for Disease Control and Prevention, Atlanta, GA, USA;

7. Eastern Europe and Central Asia Regional Office, Centers for Disease Control and Prevention, Tbilisi, Georgia;

8. National Center for Disease Control and Public Health, Tbilisi, Georgia;

9. National Center for Tuberculosis and Lung Diseases, Tbilisi, Georgia;

10. Health Research Union and Clinic NeoLab, Tbilisi, Georgia;

11. Clinic “Hepa”, Tbilisi, Georgia;

12. The University of Georgia, Tbilisi, Georgia;

13. Infectious Diseases, AIDS and Clinical Immunology Research Center, Tbilisi, Georgia;

14. Clinic “Mrcheveli”, Tbilisi, Georgia;

Corresponding author:

Davit Baliashvili (dato.baliashvili@gmail.com)

Word count: 3,985

Disclaimer: The findings and conclusions in this report are those of the authors and do not necessarily represent the official position of the US Centers for Disease Control and Prevention.

Grant Support: This work was supported in part from grants from the National Institutes of Health (NIH) Fogarty International Center (D43TW007124; "Emory-Georgia TB Research 12 Training Program") to DB and from NIH/National Institutue of Allergy and Infectious Diseases (K24AI114444; the Emory/Georgia TB Research Advancement Center, P30AI168386; the Emory University Center for AIDS Research, P30AI050409; and TB Research Unit-ASTRa, U19AI111211) to NRG.

ABSTRACT

Background: The Eastern European country of Georgia initiated a nationwide hepatitis C virus (HCV) elimination program in 2015 to address a high burden of infection. Screening for HCV infection through antibody testing was integrated into multiple existing programs, including the National Tuberculosis Program (NTP). We sought to evaluate loss to follow-up (LTFU) from the hepatitis C care cascade among persons diagnosed with active tuberculosis (TB) disease.

Methods: Using national ID numbers, we merged databases of the HCV elimination program, NTP, and national death registry from January 1, 2015, to September 30, 2020. We estimated the proportion of patients with and without TB who were LTFU at each step of the HCV care cascade and explored temporal changes.

Results: Among 11,985 patients with active TB, 9,065 (76%) were tested for HCV antibodies, and 1,665 (18%) had a positive result; LTFU from hepatitis C care was common, with 20% of patients with a positive antibody test not undergoing viremia testing, and 43% of patients with viremia not starting treatment for hepatitis C. Overall, among persons with confirmed viremic HCV infection, only 28% of patients with TB had a documented cure from HCV infection, compared to 55% among patients without TB. LTFU after positive antibody testing substantially decreased in the last three years, from 32% among patients diagnosed with TB in 2017 to 12% among those diagnosed in 2019.

Conclusions: LTFU from hepatitis C care after a positive antibody or viremia test was high and more common among patients with TB than in those without TB. Better integration of TB and hepatitis C care systems can potentially reduce LTFU and improve patient outcomes. Existing large-scale programs for both TB and hepatitis C in Georgia

create a unique opportunity for such integration to contribute to hepatitis C elimination efforts.

INTRODUCTION

Both tuberculosis (TB) and hepatitis C virus (HCV) infection cause substantial morbidity and mortality worldwide. In 2020, there were an estimated 10 million new cases of active TB globally and 1.5 million deaths due to TB [1]. There are an estimated 59 million people living with chronic HCV infection, 1.5 million new cases occur each year, and 290,000 people died due to hepatitis C globally in 2019 [2]. TB and HCV infection are often concentrated in the same high-risk population subgroups, such as homeless or incarcerated individuals and people who inject drugs (PWID) [3-5], However, the relationship between these two infectious diseases has not been well described. Specifically, it has not been well characterized how often patients with current or past TB are offered and provided with hepatitis C testing and treatment services.

Hepatitis C diagnosis, treatment, and assessment of cure consist of multiple consecutive steps, collectively known as the hepatitis C care cascade [6]. The cascade starts with hepatitis C antibody testing and ends with the determination of cure based on sustained virologic response (SVR) at 12 weeks after treatment completion [7-16]. Available data has found that loss to follow-up (LTFU) at different stages of the hepatitis C care cascade is common [17]. In 2019, globally, only 26% of people living with hepatitis C knew their infection status, and only 16% were treated [2].

The hepatitis C care cascade among patients with both TB and hepatitis C has not been previously studied. However, given the increased rate of adverse events including hepatoxicity among patients with TB and HCV coinfection, screening and potentially early treatment should be a high priority in this group [18, 19]. Historically, patients receiving treatment for TB have not been recommended to receive hepatitis C treatment

until they finish TB treatment due to drug-drug interactions [20]. However, the combined effect of TB drugs and HCV infection can cause long-term impairment of liver function, and timely management of hepatitis C in patients with TB as soon as they become eligible should be an important research priority. Since patients with TB are already receiving care, there is a key opportunity to engage them in hepatitis C care without interruption, reducing the likelihood of LTFU.

The Eastern European country of Georgia (population 3.7 million) is designated by the WHO as a high-priority country for TB control in European Region, with an estimated incidence of 70 TB cases per 100,000 population in 2020 and a high percentage of multidrug-resistant (MDR) TB [1, 21]. Chronic hepatitis C is also highly prevalent in Georgia, affecting an estimated 5.4% of the general adult population (~150,000 individuals) based on a 2015 serosurvey – the highest prevalence among countries of Eastern Europe and Central Asia [22, 23]. Historically, there has been a substantial overlap in the population affected by these two infectious diseases in Georgia; previous studies reported that >20% of newly diagnosed active TB cases had HCV antibodies, indicative of current or past infection [18, 24].

To address the high burden of HCV infection, Georgia initiated the first nationwide hepatitis C elimination program in 2015, including free hepatitis C testing and treatment with new directly-acting antivirals for all citizens [25-27]. The cost of confirmatory viremia testing was initially fully covered by the government only for persons with low income but became free for everyone beginning in March 2018 [28]. Free hepatitis C screening through antibody testing was gradually integrated into multiple existing programs and settings, including the National TB Program (NTP), which provides free

diagnostic and treatment services for TB countrywide [26, 28-31]. Before 2018, HCV antibody testing was performed routinely among patients with MDR TB and sporadically (by indication) among patients with drug-susceptible (DS) TB. According to the TB treatment guideline in Georgia adopted in July 2018, all patients newly diagnosed with TB should be routinely tested for HCV antibodies. If an antibody test is positive, blood samples are sent to the National Center for Disease Control and Public Health (NCDC) for HCV viremia testing. However, there is no formal referral system in place linking viremia-positive patients with TB to hepatitis C care.

The objectives of this study were to (1) compare the hepatitis C care cascade among patients with and without TB diagnosis in Georgia between 2015-2019; (2) identify factors associated with LTFU in hepatitis C care among patients with TB. Findings from this analysis will be a valuable contribution to the Georgian Hepatitis C Elimination Program and other similar settings by providing data for future targeted interventions to improve linkage to and retention in hepatitis C care among patients with TB.

METHODS

Study design and setting

We analyzed the hepatitis C care cascade among patients diagnosed with active TB disease and compared it to the care cascade among people with HCV without active TB disease. TB-related information was obtained from the NTP of Georgia. Hepatitis C screening, diagnostic, and treatment information was obtained from the Georgian Hepatitis C Elimination Program.

Study population

The study population consisted of two groups: 1) Adults (aged ≥18 years) diagnosed with active TB through the Georgian NTP from January 1, 2015, through December 31, 2019; and 2) Adults tested for HCV antibodies in Georgia between January 1, 2015, and September 30, 2020, who were not diagnosed with TB during that time. For both groups, hepatitis C testing and treatment information was obtained through September 30, 2020.

Hepatitis C care cascade steps and definitions

We assessed the following steps in the hepatitis C care cascade (supplemental figure 1): 1) Screening for HCV antibodies; 2) Viremia testing via ribonucleic acid (RNA) or core-antigen testing to confirm viremic HCV infection among those with HCV antibody positive result; 3) Hepatitis C treatment initiation among those with positive viremia test; 4) Hepatitis C treatment completion; 5) SVR testing from 12 to 24 weeks after treatment completion.

Two separate care cascade analyses were performed among persons with HCV infection with and without TB disease. The hepatitis C care cascade among patients with TB included the following groups: 1) Persons tested for HCV antibodies at the time of or after their first TB diagnosis; and 2) Persons tested for HCV antibodies before their TB diagnosis who did not initiate treatment for hepatitis C before their TB diagnosis, i.e. people who were eligible for hepatitis C care by the time they were enrolled in TB treatment. Hepatitis C care cascade among patients without TB included the following groups: 1) Persons tested for HCV antibodies who were not diagnosed with TB during

2015-2019; and 2) Persons diagnosed with and treated for chronic HCV infection before they were first diagnosed with TB (thus, did not have TB at the time of their HCV diagnosis and treatment).

Patients were defined as LTFU after a positive antibody test if they had a positive HCV antibody test but did not undergo HCV viremia testing within the HCV elimination program and were still alive at the time of data extraction (September 30, 2020). Patients were defined as LTFU after an HCV viremia test if they had a positive result on viremia testing (i.e., positive RNA or core antigen test) and did not start treatment for HCV infection within the HCV elimination program. Due to a lack of complete drug-susceptibility testing data, patients with TB were defined as having DR TB if they received TB treatment with second-line drugs which are used to treat MDR-TB.

Sources of data

Four data sources were utilized. We obtained all TB-related data from the Georgian NTP surveillance database. This database contains diagnostic and treatment-related information on every patient enrolled in the NTP. HCV screening information was obtained from the national HCV screening registry - a real-time, nationwide web-based system. The screening registry collects data from all sites providing HCV screening throughout the country, including TB facilities. HCV viremia testing and treatment information were obtained from the Hepatitis C Elimination Program clinical database, called "Elimination C" (ElimC) [32]. ElimC collects information related to viremia testing, other diagnostic procedures, and antiviral treatment. Patients included in any of these databases were cross-checked in the National Death Registry, and death dates were

obtained for deceased patients. A unique national ID number was used as a linking variable between all data sources.

Statistical analysis

In the care cascade analysis, we first calculated the proportions of people reaching each step of the hepatitis C care cascade, as described above. Next, we used survival analysis methods to explore if the time from a positive HCV antibody test to viremia testing and time from a positive viremia test to hepatitis C treatment initiation differed between patients with and without TB. Patients were censored at the end of the follow-up period (September 30, 2020). Death was treated as a competing risk. We used Cox proportional hazards model to calculate hazards ratios (HR) and assess the differences between groups numerically. We also created cumulative incidence curves to examine the differences graphically, and Gray's test for equality of cumulative incidence functions was used to test for differences [33, 34].

An additional analysis was performed among patients with TB to identify demographic or TB-related factors associated with LTFU in hepatitis C care after a positive antibody or viremia test. The analysis of factors associated with LTFU after a positive antibody test included all patients with TB (both DS and DR) who had a positive HCV antibody test result. The analysis of factors associated with LTFU after a positive viremia test was restricted to patients with DS TB who completed their TB treatment. Patients still on DS TB treatment were excluded because they are ineligible for hepatitis C treatment until after TB treatment is completed. Patients with DR TB were excluded due to heterogeneous TB treatment duration and lack of a standardized approach regarding the timing of

hepatitis C treatment initiation. Log-binomial regression was used to calculate adjusted risk ratios (aRR) and 95% confidence intervals (CI). Covariate selection was conducted a priori based on directed acyclic graph (DAG) theory [35]. Different sets of covariates were used for each of the patient characteristics.

Ethics

The study was approved by the institutional review boards (IRBs) at the National Center for Disease Control and Public Health (Tbilisi, Georgia), the National Center for Tuberculosis and Lung Disease (Tbilisi, Georgia), and Emory University (Atlanta, GA, USA). CDC's Human Subjects Research Office determined CDC's role to be provision of technical assistance that did not constitute engagement in the research.

RESULTS

Participants

A total of 14,993 records were obtained from the NTP database; 913 records (6.1%) were excluded due to a missing national ID number (Figure 1). The remaining 14,080 records corresponded to 12,767 individual patients, of whom 11,985 (94%) were aged ≥18 years and included in the analysis. Among those included (n=11,985), 70% were males, 82% were unemployed at the time of TB diagnosis, 6.9% reported a history of incarceration, 91% had one episode of TB treatment during the 2015-2019 period (i.e., diagnosed with TB only once), 7.9% had two episodes of TB treatment, and 1.6% had 3 to 8 episodes of TB treatment.

The hepatitis C screening registry contained data on 1,849,820 adults tested for HCV antibodies between January 1, 2015, and September 30, 2020, who were not diagnosed with TB in that period. The median age in this population was 46 years (interquartile range [IQR]: 31-62), and 54.8% were male.

Hepatitis C care cascade among persons with and without TB

After linking HCV testing information from the hepatitis C databases, 9,341 (78%) of 11,985 persons with TB were found to have been tested for HCV antibodies between January 1, 2015 – September 30, 2020. The proportion of patients with TB tested for HCV antibodies increased per year. Among patients diagnosed with TB in 2015, 60% were tested for HCV antibodies sometime during the study period. This proportion increased each subsequent year and reached 90% among patients diagnosed with TB in 2019 (Table 1). Among persons with TB included in our study, 276 (3.0%) had already started treatment for HCV infection before their first TB diagnosis and were included in the care cascade analysis of patients without TB.

Among the total of 9,065 patients with TB who were tested for hepatitis C, 1,665 (18%) patients (Figure 1) had a positive antibody test, of whom 108 (6%) died without undergoing viremia testing. Of the remaining 1,557 patients still alive, 316 (20%) were LTFU (i.e., never underwent viremia testing) (Figure 2a); among 1,241 patients who underwent viremia results, viremic HCV infection was found in 1,025 (83%) patients, of whom 443 (43%) did not start hepatitis C treatment. In a subset of patients with DR TB, the proportion of patients who were LTFU was even higher – 27% and 52% after positive antibody and viremia tests, respectively (Supplemental figure 2). For comparison, in the

hepatitis C care cascade among patients without TB (Figure 2b), the proportion of patients who were LTFU from hepatitis C care after positive antibody test or after positive viremia test were 14% and 19%, respectively. Among patients initiating HCV treatment, those with TB were also less likely to complete hepatitis C treatment (89% vs 94%) and SVR assessment (59% vs 76%) than those without TB. However, among those who completed hepatitis C treatment and were tested for SVR, the cure rate was comparable to those without TB (98.3% vs. 98.9%) (Figures 2a, 2b). Overall, among patients with confirmed viremic HCV infection, patients with TB were less likely to complete hepatitis C treatment (51%) and have a documented SVR achieved (28%), compared to patients without TB (76% and 55%, respectively; Figure 2c).

Timeliness of HCV viremia testing and treatment initiation

Among those who had information on follow-up duration available, we used survival analysis methods to compare time from positive antibody test to viremia testing or censoring between patients with TB (n=1,499) and patients without TB (n=118,396). After positive HCV antibody test, patients without TB had viremia testing sooner than patients with TB (HR = 1.46, 95% CI: 1.39, 1.54) (Figure 3); Among those who underwent viremia testing, the median time from a positive antibody test result to viremia testing was 17 days (IQR: 3-248 days) among patients with TB (n=1,116) and six days (IQR: 1-24) among patients without TB (n=97,554).

We also compared time from positive viremia test to hepatitis C treatment initiation or censoring among 1,021 patients with TB and 87,964 patients without TB who had active viremia and information on follow-up duration available. After a positive viremia test,

patients without TB started hepatitis C treatment sooner than patients with TB (HR=2.07, 95% CI: 1.91, 2.23) (Figure 4). Among those who started hepatitis C treatment, the median time from confirmation to treatment initiation was 176 days (IQR: 73-475) among patients with MDR TB (n=83), 132 days (IQR: 65-301) among patients with DS TB (n=499), and 86 days (IQR: 56-203) among patients without TB (n=71,128).

Factors associated with LTFU from hepatitis C care among patients with TB

Among patients with TB who had a positive HCV antibody test result, LTFU before viremia testing was more common among females, patients enrolled in second-line TB treatment for drug-resistant TB, and patients with unsuccessful or unknown TB treatment outcomes (Table 2). LTFU at this step of the hepatitis C care cascade substantially decreased among patients diagnosed with TB in recent years, from 32% among patients diagnosed with TB in 2017 to 12% among patients diagnosed in 2019. In multivariable analysis, MDR TB was associated with increased risk of LTFU after positive HCV antibody test (aRR=1.41, 95% CI: 1.12, 1.76).

Among patients with DS TB who had confirmed viremic HCV infection, LTFU before hepatitis C treatment initiation was more common among females, and patients with successful TB treatment outcomes (Table 3). The proportion of patients LTFU before hepatitis C treatment initiation increased by year, from 21% among patients diagnosed with TB in 2015 to 56% among patients diagnosed with TB in 2019. In multivariable analysis, we could not identify any factors meaningfully associated with LTFU before HCV treatment initiation.

DISCUSSION

In this study of two large-scale public health programs in the country of Georgia, we found that LTFU from the hepatitis C care cascade was high overall, but substantially more common among persons with TB than those without TB. Persons diagnosed with TB in 2015-2019 were found to have a high prevalence (18%) of HCV antibodies, and approximately 20% of those with HCV antibodies did not receive viremia testing to evaluate for chronic HCV infection. Furthermore, almost 2 out of 5 patients with TB who had confirmed viremic HCV infection had not started hepatitis C treatment within the National Hepatitis C Elimination program in Georgia even after they completed TB treatment. Our findings highlight the importance of improving integration and linkage to hepatitis C diagnostic and treatment services among patients with TB.

LTFU at different stages of hepatitis C care is a major barrier in controlling HCV infection globally.[17] Timely treatment of hepatitis C reduces the risk of cirrhosis, hepatocellular carcinoma, and death [36, 37]. Therefore, LTFU from hepatitis C care can increase the risk of adverse health outcomes. Additionally, untreated hepatitis C contributes to further transmission of the infection, hindering progress towards achieving elimination goals globally [17]. Georgia faces the same problem despite the nationwide Hepatitis C Elimination Program, which offers free diagnostic and treatment services. Previous studies from Georgia have found that a high proportion of patients are LTFU before viremia testing and before hepatitis C treatment initiation [26, 28]. Our findings demonstrate LTFU from hepatitis C care is even more pronounced among patients with active TB disease coinfected with HCV. Notably, among patients with TB, LTFU before HCV viremia testing has decreased in recent years, which could be explained by a change

in policy. According to Georgian 2018 TB management guideline, if an HCV antibody test is positive, blood samples are taken from the patient and sent for HCV viremia testing. Therefore, this policy change, coupled with complete financial coverage of viremia testing by the government [28], might have removed geographic, logistical, and financial barriers to viremia testing for patients with TB. This improvement highlights that systemic changes such as financial and logistic support could be key to improving retention of patients in the hepatitis C care.

Furthermore, LTFU before hepatitis C treatment initiation is also very common among patients with TB. Higher LTFU before hepatitis C treatment initiation among patients with TB compared to those without TB could be explained by the fact that TB treatment is long (from 6 to 24 months) and can be associated with severe adverse reactions [38]. Additionally, HCV treatment is usually not initiated until TB treatment completion due to drug-drug interaction concerns [20]. Therefore, in the absence of a formal linkage program, patients with TB might be more reluctant to start another long treatment course for hepatitis C due to treatment fatigue and previous negative experiences [39]. This issue has not been explicitly studied among patients with both TB and HCV. Still, fatigue from diagnostic and treatment procedures as a risk factor for treatment discontinuation has been described among patients with TB and those coinfected with human immunodeficiency virus (HIV) [40-43]. This is even more likely in the case of HCV infection without advanced liver damage because patients might not experience any symptoms associated with HCV infection and might not feel the urgency to seek hepatitis C care [44].

We also found that the proportion of patients LTFU before hepatitis C treatment initiation is increasing by year among patients who completed treatment for DS TB. Several reasons could explain this finding: 1) patients with TB diagnosed in recent years had less time to get enrolled in HCV elimination program after completing their TB treatment; 2) The COVID-19 pandemic and related restrictions in 2020 could have affected patients' ability and willingness to start treatment for hepatitis C; 3) The change in policy in 2018, as described above, removed a barrier to viremia testing for many patients, increasing the denominator of patients with TB diagnosed with HCV infection and eligible for treatment. This policy change that increased the viremia testing rates was not accompanied with a similar intervention to increase treatment initiation rates. Therefore, we can speculate that the number of patients who needed treatment (i.e., those with positive viremia result) increased at a higher rate than the number of patients who received treatment, reflected in increasing proportion of LTFU before treatment initiation in recent years. Additional surveys should be conducted among patients with TB to identify more specific reasons and challenges for not initiating hepatitis C treatment after a positive viremia test.

Results from this study have potential implications for other countries with national TB programs. In 2015, Georgia became the first country to formally initiate a nationwide hepatitis C elimination program, with significant progress achieved in scaling up HCV screening, diagnostic and treatment services throughout the country [26]. However, previously published literature and our analysis highlight that LTFU from different stages of hepatitis C care poses a major challenge to HCV elimination goals, and patients with TB are even more likely to be LTFU from hepatitis C care. To address this issue, program partners should consider introducing additional activities directed at more integrated

care of hepatitis C and TB and interventions targeted at retaining patients in hepatitis C care. These interventions could include patient navigators that have been found effective in hepatitis C linkage to care,[45] or providing hepatitis C treatment in the same facilities where they receive TB treatment. Additionally, patients with MDR TB could be treated for HCV infection and TB concomitantly. There is no documented drug-drug interaction between directly-acting antivirals used in HCV treatment and second-line TB drugs, and concomitant treatment of TB and HCV infection have been successfully implemented in small samples of patients [20, 46, 47]. For patients with DS TB, replacing rifampin with rifabutin may allow for simultaneous TB and HCV treatment [20]. Therefore, the existing infrastructure of NTP can help the HCV elimination efforts in Georgia by achieving microelimination of HCV infection among patients with TB – a strategy that is proposed as a useful approach for achieving the overall elimination goals [48].

Our study has several limitations. First, due to missing national ID numbers, we had to exclude 6% of observations from the NTP database. Second, the limited number of variables in the hepatitis C screening registry and differences in variables available in hepatitis C and TB databases did not allow us to conduct a more in-depth analysis to adjust for potential confounders. For that reason, our time-to-event analyses are limited to the crude comparison of cumulative incidence curves and crude hazards ratios between patients with and without TB, rather than trying to explore any causal associations that would require confounding adjustment. Third, we excluded patients with DR TB from the risk factor analysis of LTFU before HCV treatment initiation due to high heterogeneity in terms of when individual patients with DR TB become eligible for hepatitis C treatment.

In conclusion, we found that LTFU from hepatitis C care after positive HCV antibody and viremia testing is more common among patients with TB than those without TB. Existing large-scale public health programs for both TB and hepatitis C in Georgia create a unique opportunity for integrated care of these two infectious diseases, which could potentially reduce LTFU. Though our study was not designed to identify effective interventions for reducing LTFU, integrated care should utilize a patient-centered approach and include the following suggested interventions: 1) merging care for TB and HCV infection for coinfected patients; 2) parallel treatment of TB and hepatitis C using drug regimens without substantial drug-drug interactions; 3) active support for patients with TB to navigate the referral process and enroll in the hepatitis C treatment program, whenever it is not feasible to receive HCV care at the same facility where they receive TB treatment. These interventions can positively impact both TB and hepatitis C programs by providing timely hepatitis C care to patients with TB, thereby reducing LTFU and improving overall patient health outcomes.

ACKNOWLEDGEMENTS

The authors would like to thank Amiran Gamkrelidze and Zaza Avaliani for their support in obtaining the data used in this study.

REFERENCES

1. World Health Organization. Global Tuberculosis Report 2021. Report. Geneva: World Health Organization, 2021.

2. World Health Organization. Global progress report on HIV, viral hepatitis and sexually transmitted infections, 2021. Geneva: 2021.

3. Mehdiyev R, Alikhanova N, Gurbanova E. HIV/tuberculosis/hepatitis C virus services for incarcerated populations in Azerbaijan and the Eastern Europe Central Asia region. Curr Opin HIV AIDS. 2019;14(1):66-70. Epub 2018/11/30. doi: 10.1097/COH.0000000000000513. PubMed PMID: 30489347.

4. Beijer U, Wolf A, Fazel S. Prevalence of tuberculosis, hepatitis C virus, and HIV in homeless people: a systematic review and meta-analysis. Lancet Infect Dis. 2012;12(11):859-70. Epub 2012/08/24. doi: 10.1016/S1473-3099(12)70177-9. PubMed PMID: 22914343; PubMed Central PMCID: PMCPMC3494003.

5. Altice FL, Azbel L, Stone J, Brooks-Pollock E, Smyrnov P, Dvoriak S, et al. The perfect storm: incarceration and the high-risk environment perpetuating transmission of HIV, hepatitis C virus, and tuberculosis in Eastern Europe and Central Asia. Lancet. 2016;388(10050):1228-48. Epub 2016/07/19. doi: 10.1016/S0140-6736(16)30856-X. PubMed PMID: 27427455; PubMed Central PMCID: PMCPMC5087988.

6. Safreed-Harmon K, Blach S, Aleman S, Bollerup S, Cooke G, Dalgard O, et al. The Consensus Hepatitis C Cascade of Care: Standardized Reporting to Monitor Progress Toward Elimination. Clin Infect Dis. 2019;69(12):2218-27. Epub 2019/07/29. doi: 10.1093/cid/ciz714. PubMed PMID: 31352481.

7. Strader DB, Wright T, Thomas DL, Seeff LB, American Association for the Study of Liver D. Diagnosis, management, and treatment of hepatitis C. Hepatology. 2004;39(4):1147-71. Epub 2004/04/02. doi: 10.1002/hep.20119. PubMed PMID: 15057920.

8. Peeling RW, Boeras DI, Marinucci F, Easterbrook P. The future of viral hepatitis testing: innovations in testing technologies and approaches. BMC infectious diseases. 2017;17(Suppl 1):699. Epub 2017/11/17. doi: 10.1186/s12879-017-2775-0. PubMed PMID: 29143676; PubMed Central PMCID: PMCPMC5688478.

9. Guidelines for the Care and Treatment of Persons Diagnosed with Chronic Hepatitis C Virus Infection. WHO Guidelines Approved by the Guidelines Review Committee. Geneva2018.

10. Popping S, Bade D, Boucher C, van der Valk M, El-Sayed M, Sigurour O, et al. The global campaign to eliminate HBV and HCV infection: International Viral Hepatitis Elimination Meeting and core indicators for development towards the 2030 elimination goals. J Virus Erad. 2019;5(1):60-6. Epub 2019/02/26. PubMed PMID: 30800429; PubMed Central PMCID: PMCPMC6362901.

11. In: Buckley GJ, Strom BL, editors. Eliminating the Public Health Problem of Hepatitis B and C in the United States: Phase One Report. Washington (DC)2016.

12. J. Dore G, Marianne Martinello, Maryam Alavi, Jason Grebely. Global elimination of hepatitis C virus by 2030: why not? 2020;26:157-60. doi: 10.1038/s41591-019-0706-x.

13. Spengler U. Direct antiviral agents (DAAs) - A new age in the treatment of hepatitis C virus infection. Pharmacol Ther. 2018;183:118-26. Epub 2017/10/13. doi: 10.1016/j.pharmthera.2017.10.009. PubMed PMID: 29024739.

14. Li HC, Lo SY. Hepatitis C virus: Virology, diagnosis and treatment. World journal of hepatology. 2015;7(10):1377-89. Epub 2015/06/09. doi: 10.4254/wjh.v7.i10.1377. PubMed PMID: 26052383; PubMed Central PMCID: PMCPMC4450201.

15. Dore GJ, Feld JJ. Hepatitis C virus therapeutic development: in pursuit of "perfectovir". Clin Infect Dis. 2015;60(12):1829-36. Epub 2015/03/13. doi: 10.1093/cid/civ197. PubMed PMID: 25761867.

16. Gretch DR. Use and interpretation of HCV diagnostic tests in the clinical setting. Clin Liver Dis. 1997;1(3):543-57, vi. Epub 2004/11/25. doi: 10.1016/s1089-3261(05)70320-2. PubMed PMID: 15560057.

17. Dijk M, Drenth JPH, Arends JE, Brakenhoff SM, Isfordink CJ, De Knegt R, et al. Loss to follow- up in the hepatitis C care cascade: A substantial problem but opportunity for micro-elimination. Journal of Viral Hepatitis. 2020;27(12):1270-83. doi: 10.1111/jvh.13399.

18. Lomtadze N, Kupreishvili L, Salakaia A, Vashakidze S, Sharvadze L, Kempker RR, et al. Hepatitis C virus co-infection increases the risk of anti-tuberculosis drug-induced hepatotoxicity among patients with pulmonary tuberculosis. PloS one. 2013;8(12):e83892. Epub 2013/12/25. doi: 10.1371/journal.pone.0083892. PubMed PMID: 24367617; PubMed Central PMCID: PMCPMC3868578.

19. Chang TE, Huang YS, Chang CH, Perng CL, Huang YH, Hou MC. The susceptibility of anti-tuberculosis drug-induced liver injury and chronic hepatitis C infection: A systematic review and meta-analysis. J Chin Med Assoc. 2018;81(2):111-8. Epub 2017/12/05. doi: 10.1016/j.jcma.2017.10.002. PubMed PMID: 29198550.

20. Kempker RR, Alghamdi WA, Al-Shaer MH, Burch G, Peloquin CA. A Pharmacology Perspective on Simultaneous Tuberculosis and Hepatitis C Treatment. Antimicrob Agents Chemother. 2019;63(12). doi: 10.1128/aac.01215-19. PubMed PMID: 31591118.

21. World Health Organization. Roadmap to implement the tuberculosis action plan for the WHO European Region 2016–2020. 2016.

22. Maistat L, Kravchenko N, Reddy A. Hepatitis C in Eastern Europe and Central Asia: a survey of epidemiology, treatment access and civil society activity in eleven countries. Hepatol Med Policy. 2017;2. doi: 10.1186/s41124-017-0026-z. PubMed PMID: 30288322.

23. Hagan LM, Kasradze A, Salyer SJ, Gamkrelidze A, Alkhazashvili M, Chanturia G, et al. Hepatitis C prevalence and risk factors in Georgia, 2015: setting a baseline for elimination. BMC Public Health. 2019;19(3):480. doi: 10.1186/s12889-019-6784-3.

24. Richards DC, Mikiashvili T, Parris JJ, Kourbatova EV, Wilson JC, Shubladze N, et al. High prevalence of hepatitis C virus but not HIV co-infection among patients with tuberculosis in Georgia. Int J Tuberc Lung Dis. 2006;10(4):396-401. PubMed PMID: 16602403.

25. Mitruka K, Tsertsvadze T, Butsashvili M, Gamkrelidze A, Sabelashvili P, Adamia E, et al. Launch of a Nationwide Hepatitis C Elimination Program--Georgia, April 2015. MMWR Morb Mortal Wkly Rep. 2015;64(28):753-7. Epub 2015/07/24. PubMed PMID: 26203628; PubMed Central PMCID: PMCPMC4584859.

26. Averhoff F, Lazarus JV, Sergeenko D, Colombo M, Gamkrelidze A, Tsertsvadze T, et al. Excellence in Viral Hepatitis Elimination - Lessons from Georgia. J Hepatol. 2019. Epub 2019/07/30. doi: 10.1016/j.jhep.2019.06.026. PubMed PMID: 31356831.

27. Tsertsvadze T, Gamkrelidze A, Chkhartishvili N, Abutidze A, Sharvadze L, Kerashvili V, et al. Three years of progress towards achieving hepatitis C elimination in the country of

Georgia, April 2015 - March 2018. Clin Infect Dis. 2019. Epub 2019/09/30. doi: 10.1093/cid/ciz956. PubMed PMID: 31563938.

28. Averhoff F, Shadaker S, Gamkrelidze A, Kuchuloria T, Gvinjilia L, Getia V, et al. Progress and Challenges in a Pioneering Hepatitis C Elimination Program in the Country of Georgia, 2015-2018. J Hepatol. 2019. Epub 2019/12/08. doi: 10.1016/j.jhep.2019.11.019. PubMed PMID: 31811882.

29. Nasrullah M, Sergeenko D, Gvinjilia L, Gamkrelidze A, Tsertsvadze T, Butsashvili M, et al. The Role of Screening and Treatment in National Progress Toward Hepatitis C Elimination - Georgia, 2015-2016. MMWR Morb Mortal Wkly Rep. 2017;66(29):773-6. Epub 2017/07/28. doi: 10.15585/mmwr.mm6629a2. PubMed PMID: 28749925; PubMed Central PMCID: PMCPMC5657814.

30. Bloch EM, Kipiani E, Shadaker S, Alkhazashvili M, Gvinjilia L, Kuchuloria T, et al. Blood transfusion safety in the country of Georgia: collateral benefit from a national hepatitis C elimination program. Transfusion. n/a(n/a). doi: 10.1111/trf.15815.

31. Stvilia K, Spradling PR, Asatiani A, Gogia M, Kutateladze K, Butsashvili M, et al. Progress in Testing for and Treatment of Hepatitis C Virus Infection Among Persons Who Inject Drugs - Georgia, 2018. MMWR Morb Mortal Wkly Rep. 2019;68(29):637-41. Epub 2019/07/26. doi: 10.15585/mmwr.mm6829a2. PubMed PMID: 31344021; PubMed Central PMCID: PMCPMC6660105 potential conflicts of interest. No potential conflicts of interest were disclosed.

32. Shadaker S, Nasrullah M, Gamkrelidze A, Ray J, Gvinjilia L, Kuchuloria T, et al. Screening and linkage to care for hepatitis C among inpatients in Georgia's national hospital screening program. Prev Med. 2020;138:106153. Epub 2020/05/31. doi:

10.1016/j.ypmed.2020.106153. PubMed PMID: 32473265; PubMed Central PMCID: PMCPMC7440391.

33. Dignam JJ, Kocherginsky MN. Choice and Interpretation of Statistical Tests Used When Competing Risks Are Present. Journal of Clinical Oncology. 2008;26(24):4027-34. doi: 10.1200/jco.2007.12.9866.

34. Gray RJ. A class of K-sample tests for comparing the cumulative incidence of a competing risk. The Annals of Statistics. 1988;16(3):1141-54.

35. Greenland S, Pearl J, Robins JM. Causal diagrams for epidemiologic research. Epidemiology. 1999;10(1):37-48. Epub 1999/01/15. PubMed PMID: 9888278.

36. Su F, Ioannou GN. The impact of direct-acting antiviral therapy for hepatitis C on hepatocellular carcinoma risk. Curr Hepatol Rep. 2018;17(4):377-84. Epub 20180920. doi: 10.1007/s11901-018-0424-8. PubMed PMID: 30923667; PubMed Central PMCID: PMCPMC6433385.

37. Backus LI, Belperio PS, Shahoumian TA, Mole LA. Direct-acting antiviral sustained virologic response: Impact on mortality in patients without advanced liver disease. Hepatology. 2018;68(3):827-38. Epub 20180514. doi: 10.1002/hep.29811. PubMed PMID: 29377196.

38. Lan Z, Ahmad N, Baghaei P, Barkane L, Benedetti A, Brode SK, et al. Drug-associated adverse events in the treatment of multidrug-resistant tuberculosis: an individual patient data meta-analysis. Lancet Respir Med. 2020;8(4):383-94. Epub 2020/03/21. doi: 10.1016/S2213-2600(20)30047-3. PubMed PMID: 32192585.

39. Aibana O, Dauria E, Kiriazova T, Makarenko O, Bachmaha M, Rybak N, et al. Patients' perspectives of tuberculosis treatment challenges and barriers to treatment adherence in

Ukraine: a qualitative study. BMJ Open. 2020;10(1). doi: 10.1136/bmjopen-2019-032027. PubMed PMID: 32014870.

40. Skinner D, Claassens M. It's complicated: why do tuberculosis patients not initiate or stay adherent to treatment? A qualitative study from South Africa. BMC infectious diseases. 162016.

41. O'Donnell MR, Padayatchi N, Daftary A, Orrell C, Dooley KE, Amico KR, et al. Antiretroviral switching and bedaquiline treatment of drug-resistant tuberculosis HIV co-infection. Lancet HIV. 2019;6(3):e201-4. doi: 10.1016/s2352-3018(19)30035-9. PubMed PMID: 30846058; PubMed Central PMCID: PMC7155388.

42. O'Donnell MR, Daftary A, Frick M, Hirsch-Moverman Y, Amico KR, Senthilingam M, et al. Re-inventing adherence: toward a patient-centered model of care for drug-resistant tuberculosis and HIV. Int J Tuberc Lung Dis. 2016;20(4):430-4. doi: 10.5588/ijtld.15.0360. PubMed PMID: 26970149; PubMed Central PMCID: PMC4863995.

43. Furin J, Isaakidis P, Reid AJ, Kielmann K. 'I'm fed up': experiences of prior anti-tuberculosis treatment in patients with drug-resistant tuberculosis and HIV. Int J Tuberc Lung Dis. 2014;18(12):1479-84. Epub 2014/12/18. doi: 10.5588/ijtld.14.0277. PubMed PMID: 25517815.

44. Westbrook RH, Dusheiko G. Natural history of hepatitis C. J Hepatol. 2014;61(1 Suppl):S58-68. Epub 2014/12/03. doi: 10.1016/j.jhep.2014.07.012. PubMed PMID: 25443346.

45. Strebe J, Rich NE, Wang L, Singal AG, McBryde J, Silva M, et al. Patient Navigation Increases Linkage to Care and Receipt of Direct-Acting Antiviral Therapy in Patients with Hepatitis C. Clin Gastroenterol Hepatol. 2022. Epub 20220513. doi: 10.1016/j.cgh.2022.04.031. PubMed PMID: 35577048.

46. Melikyan N, Huerga H, Atshemyan H, Kirakosyan O, Sargsyants N, Aydinyan T, et al. Concomitant Treatment of Chronic Hepatitis C With Direct-Acting Antivirals and Multidrug-Resistant Tuberculosis Is Effective and Safe. Open Forum Infectious Diseases. 2021;8(2). doi: 10.1093/ofid/ofaa653.

47. Tunesi S, Du DL, Gualano G, Millet JP, Skrahin A, Bothamley G, et al. Co-administration of treatment for rifampicin-resistant TB and chronic HCV infection: A TBnet and ESGMYC study. The Journal of infection. 2022;84(6):834-72. Epub 20220307. doi: 10.1016/j.jinf.2022.03.004. PubMed PMID: 35271917.

48. Safreed-Harmon K, Thursz M, Dillon J, El-Sayed M, Elsharkawy A, Hatzakis A, et al. The Micro-Elimination Approach to Eliminating Hepatitis C: Strategic and Operational Considerations. Seminars in Liver Disease. 2018;38(03):181-92. doi: 10.1055/s-0038-1666841.

TABLES

Table 1. HCV testing by year of first TB diagnosis among adult patients diagnosed with TB in 2015-2019.

	Patients diagnosed with TB	Ever Tested for HCV antibodies[a]		First HCV antibody testing before or during the last TB episode		First HCV antibody testing after last TB episode outcome		Ever tested positive for HCV antibodies	
Year	N	N	%	N	% (of total ever tested)	N	% (of total ever tested)	N	%
2015	2766	1671	60%	485	29%	1186	71%	414	25%
2016	2740	1860	68%	855	46%	1005	54%	410	22%
2017	2405	1948	81%	1610	83%	338	17%	333	17%
2018	2086	1791	86%	1635	91%	156	9%	255	14%
2019	1988	1795	90%	1773	99%	22	1%	253	14%

[a] excluding patients who started treatment for HCV infection before first TB diagnosis

Abbreviation: HCV, hepatitis C virus; TB, tuberculosis

Table 2. Loss to follow-up from HCV care before HCV viremia testing among patients with TB.

CHARACTERISTICS (based on first TB diagnosis)	Total (N=1557) N	% (col)	LTFU (n=318) N	% (row)	Not LTFU (n=1239) N	% (row)	cRR (95%CI)	aRR (95%CI)
**Sex**								
Male	1411	91%	273	19%	1138	81%	1	
Female	146	9%	45	31%	101	69%	1.59 (1.22, 2.08)	
**Age**								
Median (IQR)	45 (16)		43 (16)		46 (16)			
**Region**								
Tbilisi	620	40%	138	22%	482	78%	1	
Other	885	57%	173	20%	712	80%	0.88 (0.72, 1.07)	
Penitentiary system	52	3%	7	13%	45	87%	0.60 (0.30, 1.22)	
**Employment status**								
Employed	155	10%	34	22%	121	78%	1	1
Unemployed	1344	86%	276	21%	1068	79%	0.94 (0.68, 1.28)	0.95 (0.69, 1.30)[a]
Military	2	0%	0	0%	2	100%	-	
Missing	56	4%	8	14%	48	86%	-	
**IDP**								
Yes	131	8%	37	28%	94	72%	1.44 (1.07, 1.93)	1.38 (1.04, 1.85)[b]
No	1342	86%	263	20%	1079	80%	1	1
Missing	84	5%	18	21%	66	79%	-	
**History of imprisonment**								
Yes	295	19%	55	19%	240	81%	0.91 (0.70, 1.18)	
No	1192	77%	245	21%	947	79%	1	
Missing	70	4%	18	26%	52	74%	-	
**TB DIAGNOSIS AND TREATMENT**								

**Year of TB diagnosis**								
2015	386	25%	64	17%	322	83%	1.35 (0.90, 2.02)	
2016	374	24%	76	20%	298	80%	1.65 (1.12, 2.44)	
2017	304	20%	97	32%	207	68%	2.60 (1.79, 3.77)	
2018	249	16%	51	20%	198	80%	1.67 (1.10, 2.52)	
2019	244	16%	30	12%	214	88%	1	
**Newly diagnosed TB**								
Yes	1066	68%	217	20%	849	80%	1	1
No/Unknown	491	32%	101	21%	390	79%	1.01 (0.82, 1.25)	1.07 (0.86, 1.34)[b]
**MDR TB**								
Yes	277	18%	75	27%	202	73%	1.43 (1.14, 1.78)	1.41 (1.12, 1.76)[c]
No	1280	82%	243	19%	1037	81%	1	1
**Treatment outcome**								
Successful	985	63%	184	19%	801	81%	1	1
Unsuccessful/Unknown	509	33%	125	25%	384	75%	1.31 (1.08, 1.61)	1.10 (0.84, 1.45)[d]
Missing	63	4%	9	14%	54	86%	-	

Note: Study population in this analysis included patients diagnosed with TB in 2015-2019 who had a positive HCV antibody test result. Comparison groups: 1) Patients with TB who did not undergo viremia testing and are still alive (LTFU) and, 2) Patients with TB who underwent HCV viremia testing (not LTFU). HCV viremia testing status ascertained as of September 30, 2020. Patients with positive HCV antibody test who died are excluded from this analysis.

Abbreviations: TB, tuberculosis; HCV, hepatitis C virus; LTFU, loss to follow-up; cRR, crude risk ratio; aRR, adjusted risk ratio; IQR, interquartile range; IDP, internally displaced person; Tx, treatment; MDR, multidrug-resistant TB.

[a] adjusted for age, sex, IDP, MDR TB, case definition (new vs. previously treated);

[b] adjusted for age, sex, MDR TB, employment status;

[c] adjusted for age, sex, case definition (new vs. previously treated);

[d] adjusted for age, sex, MDR TB, employment status;

Table 3. Loss to follow-up from hepatitis C care before treatment initiation among patients with drug-susceptible TB.

CHARACTERISTICS (based on first TB diagnosis)	Total (N=758) N	%	LTFU (n=259) N	%	Not LTFU (n=499) N	%	cRR (95%CI)	aRR (95%CI)
**DEMOGRAPHIC CHARACTERISTICS**								
**Sex**								
Male	694	92%	230	33%	464	67%	1	
Female	64	8%	29	45%	35	55%	1.37 (1.02, 1.83)	
**Age**								
Median (IQR)	45 (15)		46 (16)		44 (14)			
**Region**								
Tbilisi	290	38%	95	33%	195	67%	1	
Other	441	58%	157	36%	284	64%	1.09 (0.88, 1.34)	
Penitentiary system	27	4%	7	26%	20	74%	0.79 (0.41, 1.53)	
**Employment status**								
Employed	75	10%	25	33%	50	67%	1	
Unemployed	647	85%	218	34%	429	66%	1.01 (0.72, 1.42)	0.93 (0.67, 1.30)[a]
Military	2	0%	1	50%	1	50%	-	
Missing	34	4%	15	44%	19	56%	-	
**IDP**								
Yes	67	9%	22	33%	45	67%	0.97 (0.68, 1.39)	
No	655	86%	221	34%	434	66%	1	
Missing	36	5%	16	44%	20	56%		
**History of imprisonment**								
Yes	597	79%	47	36%	84	64%	1.05 (0.81, 1.35)	
No	131	17%	204	34%	393	66%	1	
Missing	30	4%	8	27%	22	73%		
**TB DIAGNOSIS AND TREATMENT**								

**Year of TB diagnosis**								
2015	193	25%	41	21%	152	79%	1	
2016	199	26%	46	23%	153	77%	1.09 (0.75, 1.58)	
2017	135	18%	47	35%	88	65%	1.64 (1.15, 2.34)	
2018	117	15%	61	52%	56	48%	2.45 (1.78, 3.39)	
2019	114	15%	64	56%	50	44%	2.64 (1.93, 3.63)	
**# of TB Tx episodes (binary)**								
1	647	85%	231	36%	416	64%	1	
2+	111	15%	28	25%	83	75%	0.71 (0.50, 0.99)	
**Newly diagnosed TB**								
Yes	555	73%	193	35%	362	65%	1	1
No/Unknown	203	27%	66	33%	137	67%	0.93 (0.74, 1.18)	0.92 (0.72, 1.16)[b]
**Treatment outcome (binary)**								
Successful	611	81%	214	35%	397	65%	1.09 (0.84, 1.42)	1.04 (0.79, 1.38)[c]
Unsuccessful/Unknown	137	18%	44	32%	93	68%	1	1
Missing	10	1%	1	10%	9	90%	-	

Note: Study population included in this table include patients diagnosed with drug-susceptible TB in 2015-2019 who have confirmed HCV infection (positive viremia test). Comparison groups: 1), Patients with TB who are eligible but did not start HCV treatment as of September 2020 (LTFU), and 2) Patients with TB who started HCV treatment (not LTFU). Patients with positive HCV viremia test who died or are still on TB treatment are excluded from this analysis.

Abbreviations: TB, tuberculosis; HCV, hepatitis C virus; LTFU, loss to follow-up; cRR, crude risk ratio; aRR, adjusted risk ratio; IQR, interquartile range; IDP, internally displaced person; Tx, treatment;

[a] adjusted for age, sex, IDP, case definition (new vs. previously treated);

[b] adjusted for age, sex, employment status;

[c] adjusted for age, sex, employment status;

FIGURES

Figure 1. Flow chart of patients with TB and their HCV screening status - 2015-2019

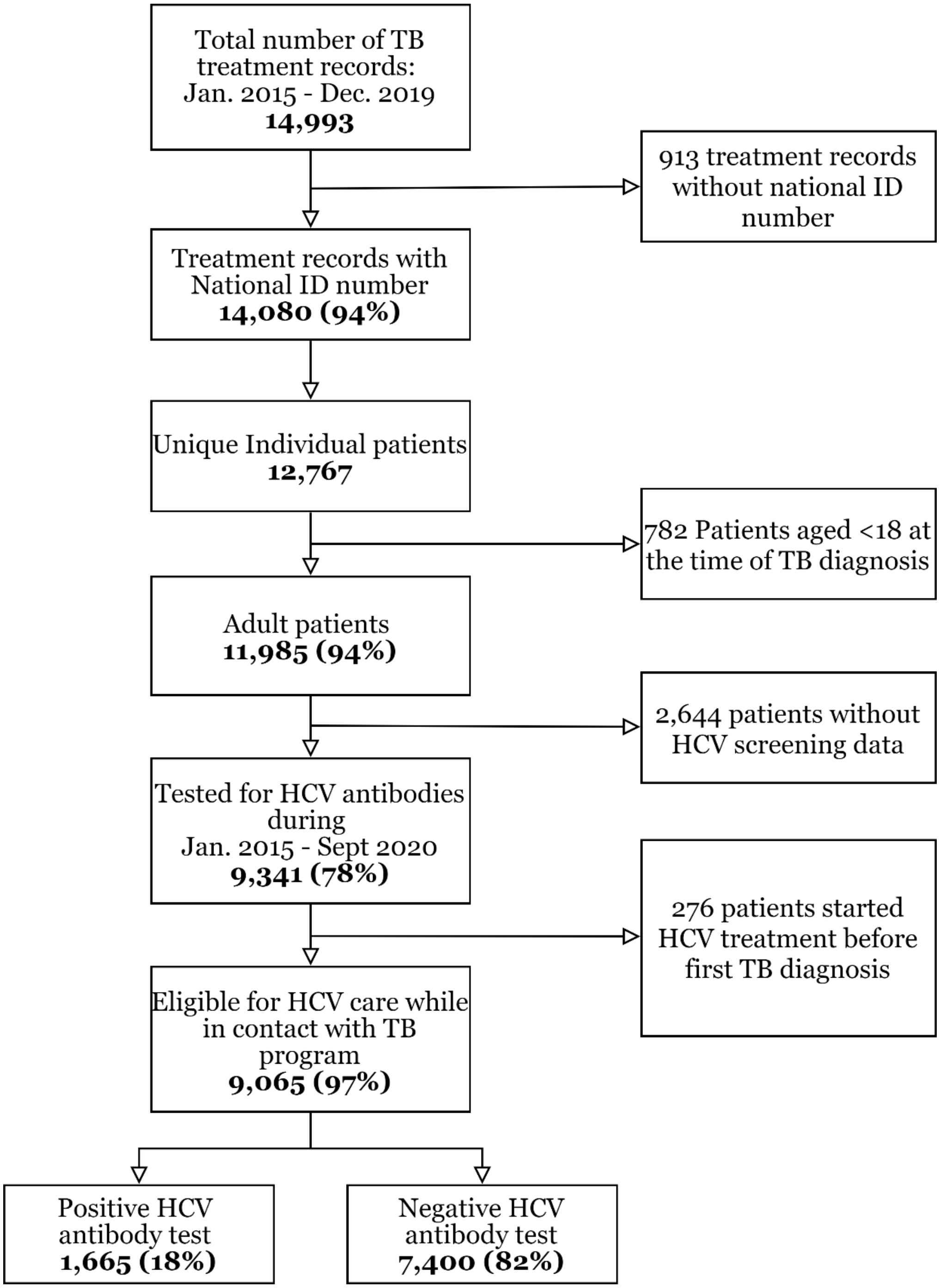

Abbreviation: HCV, hepatitis C virus; TB, tuberculosis;

Figure 2. Hepatitis C care cascade among patients with and without tuberculosis. a. Hepatitis C cascade of care among HCV seropositive patients diagnosed with TB in 2015-2019; b. Hepatitis C cascade of care among HCV seropositive patients without TB diagnosis in 2015-2019; c. Cumulative proportions of patients reaching each step of cascade, out of total number of patients with active HCV infection

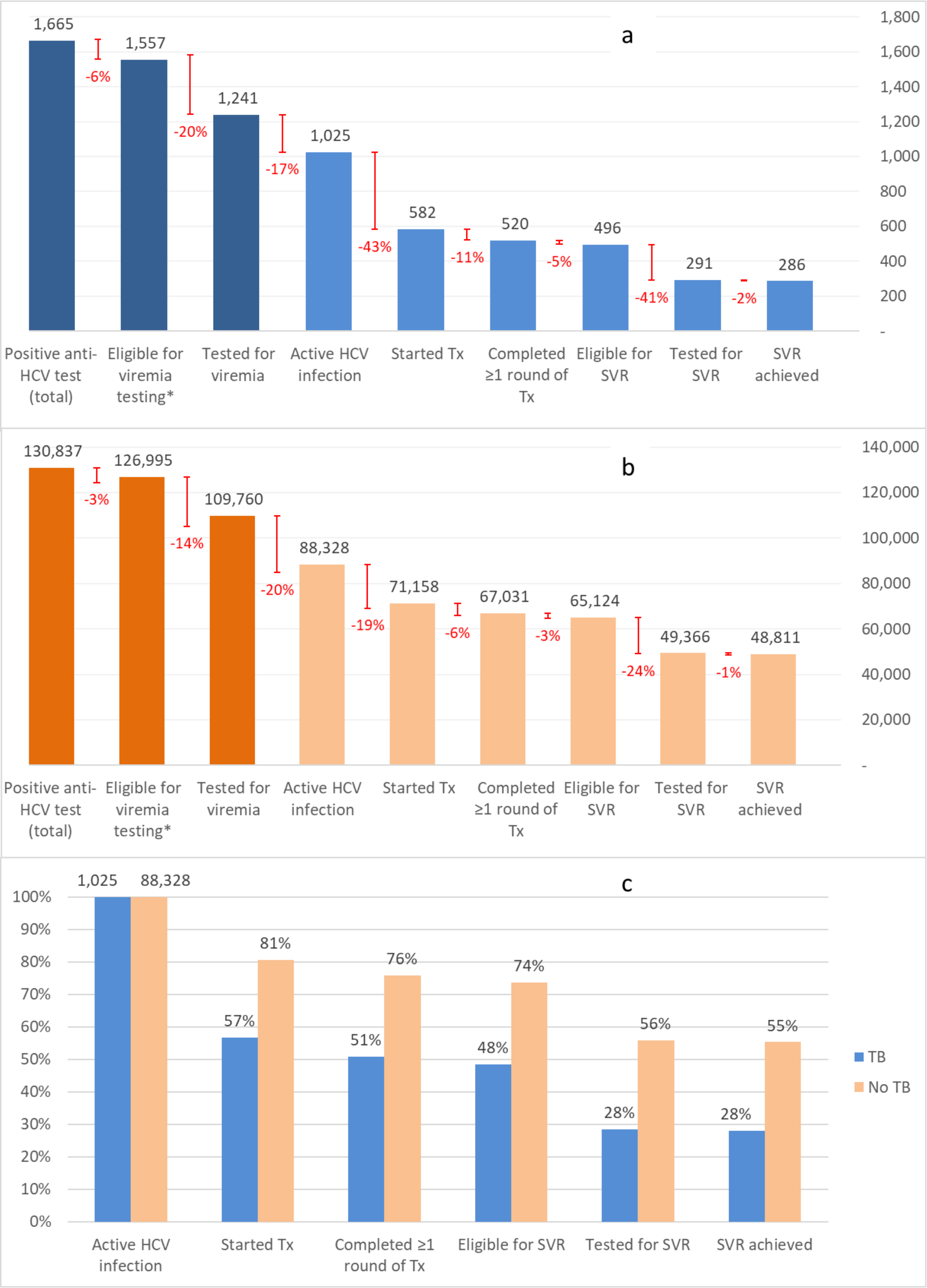

* No death before viremia testing

Note: bars in dark blue and dark orange in panels a and b are not included in panel c.

Abbreviation: HCV, hepatitis C virus; TB, tuberculosis; SVR, sustained virologic response.

Figure 3. Comparison of HCV viremia testing cumulative incidence among HCV seropositive patients with and without TB[a,b]: January 1, 2015 – September 30, 2020.

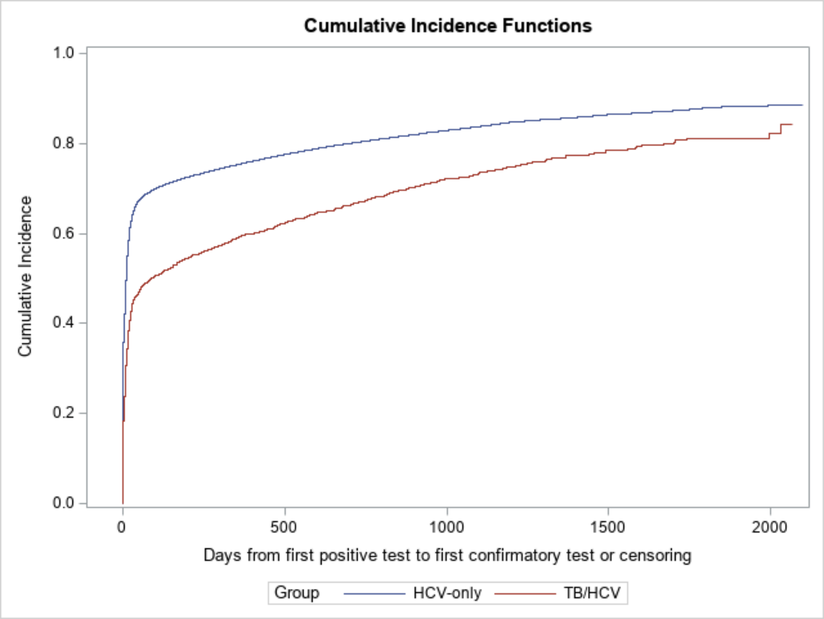

[a] Blue line corresponds to the patients with confirmed HCV infection without evidence of TB diagnosis in 2015-2019. Red line corresponds to patients with confirmed HCV infection diagnosed with TB in 2015-2019.

[b] Gray's test p-value<0.001.

Figure 4. Comparison of cumulative incidence of HCV treatment initiation among patients with confirmed HCV infection with and without TB[a,b]: January 1, 2015 – September 30, 2020.

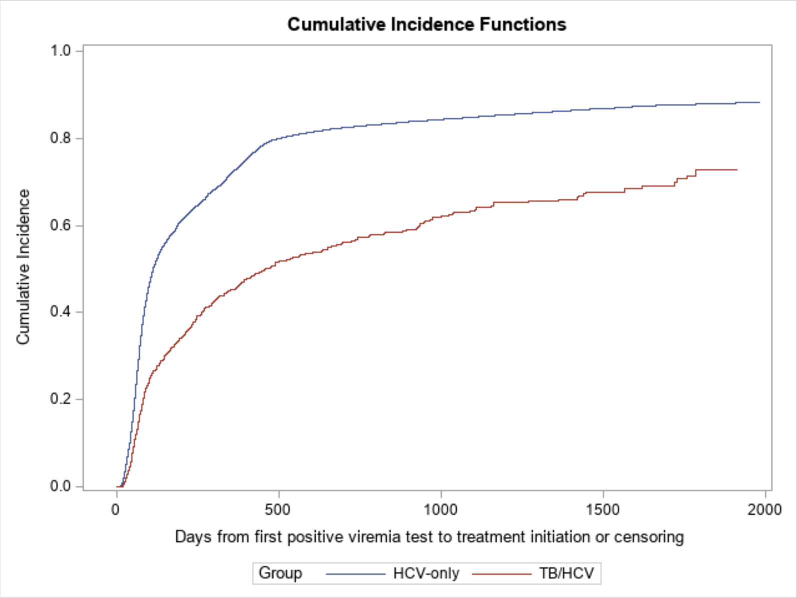

[a] Blue line corresponds to patients with confirmed HCV infection without evidence of TB diagnosis in 2015-2019. Red line corresponds to patients with confirmed HCV infection diagnosed with TB in 2015-2019.

[b] Gray's test p-value<0.001

Click here to access/download
Supporting Information
Supplemental material.docx
