# Supplementary material for: Hepatitis C care cascade among patients with and without tuberculosis: Nationwide observational cohort study in the country of Georgia, 2015–2020
Source: PLoS Med. 2023 May 4;20(5):e1004121. doi: 10.1371/journal.pmed.1004121 (PMC10194957; doi:10.1371/journal.pmed.1004121)
Supplement: Attachment — Submitted filename: Supplemental material_review.docx [file pmed.1004121.s015.docx]

**Supplemental material for the manuscript “Hepatitis C care cascade among patients with and without tuberculosis: findings from nationwide programs in the country of Georgia, 2015-2020”**

**Figure S1**. Steps of hepatitis C care cascade in Georgia.

**Figure S2**. Hepatitis C care cascade among patients with TB, stratified by their drug-resistance status

* No death before viremia testing
