# Supplementary material for: Hepatitis C care cascade among patients with and without tuberculosis: Nationwide observational cohort study in the country of Georgia, 2015–2020
Source: PLoS Med. 2023 May 4;20(5):e1004121. doi: 10.1371/journal.pmed.1004121 (PMC10194957; doi:10.1371/journal.pmed.1004121)
Supplement: Attachment — Submitted filename: Response to reviewers.docx [file pmed.1004121.s016.docx]

Dear editors and reviewers,

The authors of the manuscript thank you for reviewing this manuscript and providing constructive feedback. We believe that addressing your comments improved the quality of our manuscript greatly. We appreciate your time spent reviewing the original and this revised version of the manuscript. We thank you for considering this manuscript for publishing in a highly respected journal such as Plos Medicine.

Below, we are responding to each comment the editor and reviewers provided. To ease of navigating between comments and referencing the similar comments already addressed, we numbered the comments consecutively, starting from the editor’s comments. Whenever we changed the manuscript text in response to the comment, we included the line numbers corresponding to the lines in the tracked version of the manuscript file.

We look forward to hearing your decision or further feedback.

Best regards,

Davit Baliashvili

**Comments from the editorial office:**

**1. Comment**: Please ensure that the study is reported according to the STROBE guideline, and include the completed STROBE checklist as Supporting Information.  Please add the following statement, or similar, to the Methods: "This study is reported as per the Strengthening the Reporting of Observational Studies in Epidemiology (STROBE) guideline (S1 Checklist)."
The STROBE guideline can be found here: <http://www.equator-network.org/reporting-guidelines/strobe/>
When completing the checklist, please use section and paragraph numbers, rather than page numbers.

**Response**: We reviewed the manuscript to ensure the study is reported according to the STROBE guideline. We include a completed STROBE checklist with this revised manuscript. As requested by the editor, we added the text in the methods section stating that the study is reported according to the STROBE guideline. (Lines 274-276)

**COMMENTS FROM THE ACADEMIC EDITOR**
There have been several papers looking at HCV treatment cascades, although most come from Europe/US among selected populations or among other target populations (IVDU and/or incarcerated persons). Although TB-positive patients are perhaps less relevant to US and parts of Europe, this is important from international perspective and several countries with remaining HCV pockets. With that in mind, I agree reasonable to proceed with major revision. In addition to comments from reviewers, I had some thoughts I listed below:

**2. Comment** 1. Greater details regarding any interventions that were put in place to promote linkage to care (even as simple as local or national education campaigns).

**Response**: We expanded the paragraph in the discussion section describing the LTFU issue in Georgia and mentioned one ongoing intervention that was put in place to actively trace those who are LTFU and reengage them in care. However, we also mention that similar activities specifically targeted at people with TB have not been introduced (lines 415-423). We are unaware of any other interventions in Georgia aimed at improving the linkage to care that have been implemented.

**3. Comment** 2. There was improvement in HCV Ab testing over time; was there changes in follow-up testing over time?

**Response**: Confirmatory testing was centralized starting in 2018. A blood sample was taken and sent to a national reference laboratory for confirmatory testing if a patient with TB tested positive for HCV antibodies. We describe this process in the discussion section (lines 426-428). Besides this, there have not been any other changes in terms of follow-up testing

**4. Comment** 3. Patients with active HCV who failed to get treated could use more detail. Were these patients referred for care and/or seen in clinic?

**Response**: We added further details regarding the barriers to getting HCV treatment in the discussion section (lines 415-423). We do not have all the answers as to why patients with active HCV infection fail to get the treatment, but we acknowledge the importance of this question as an important area to explore further. Previously the cost related to copayment for the confirmatory testing was considered a primary barrier, but it has been eliminated since then, given that there are no copayments anymore and the national program fully covers all procedures. It is challenging to describe the exact reasons why the patients were not treated in recent years. We suspect it may be from a combination of personal perceptions about the treatment, mistrust towards the national treatment program, lack of perceived threat due to the asymptomatic nature of the disease, and other unidentified reasons. Unfortunately, there have not been any more recent studies of the barriers to elucidate the main reasons for LTFU before treatment. As for whether they are referred for care and seen in the clinic, this also differs by the setting. If the confirmatory testing is done in a specialized clinic, patients are usually consulted by a clinician. In some settings, such as TB clinics, after positive antibody testing, the samples are taken and sent to a central laboratory so the patient does not have to go the hepatitis C service provider clinic to get a diagnosis. This has improved the proportion of patients with confirmatory testing, but the proportion of LTFU after hepatitis C diagnosis also increased. This might be partly explained by the fact that TB patients do not see hepatitis C service providers for their viremia diagnosis and thus might be less informed about the need of the treatment. We describe the intricacies of the viremia testing and treatment initiation among patients with TB in the Discussion section lines 426-428 and 453-460.

**5. Comment** 4. Do authors have any data regarding comorbidity or life expectancy of patients? Some of the cascade failures may have been appropriate lack of referrals/treatment given comorbidities, including decompensated cirrhosis (Child C cirrhosis) where HCV treatment is expected to have less impact.

**Response**: Unfortunately, we do not have detailed data on comorbidities. In the Georgian hepatitis C elimination program, a detailed patient assessment, including liver fibrosis staging, is conducted when a patient is enrolled at the beginning of treatment. Therefore, people who are lost to follow-up do not have that information recorded in the dataset. However, it is worth mentioning that decompensated cirrhosis was not a reason for the lack of referral or treatment by the design of elimination program. Conversely, at the beginning of the program, people with advanced liver fibrosis, including decompensated cirrhosis, were prioritized for treatment. Since then, the treatment eligibility has expanded, and everyone with any stage of liver fibrosis is eligible to get treatment within the program. Below, we provide the reference to the paper describing the treatment eligibility and outcomes in the initial stage of the elimination program:

Tengiz Tsertsvadze, Amiran Gamkrelidze, Muazzam Nasrullah, Lali Sharvadze, Juliette Morgan, Shaun Shadaker, Lia Gvinjilia, Maia Butsashvili, David Metreveli, Vakhtang Kerashvili, Marina Ezugbaia, Nikoloz Chkhartishvili, Akaki Abutidze, Valeri Kvaratskhelia and Francisco Averhoff. Treatment outcomes of patients with chronic hepatitis C receiving sofosbuvir-based combination therapy within national hepatitis C elimination program in the country of Georgia. BMC Infect Dis. 2020 Jan 10;20(1):30. doi: 10.1186/s12879-019-4741-5.

**6. Comment** 5. The TB and non-TB populations are inherently different but I did not see characteristics of the patients, stratified by TB status to detail those differences. To that end, I question if a propensity-score matched analysis would be possible/informative.

**Response**: We agree with the reviewer that the population of patients with and without TB likely differ. To provide more details about these populations, we submit three supplemental tables (Tables S1, S2, S3) with baseline characteristics for each of these groups that were available in our datasets. We would like to clarify that in the first objective of our study when we compare the patients with and without TB and their care cascade, we do not aim to make any causal claims about whether TB causes LTFU from hepatitis C care. We merely want to describe the gaps across the care cascade and compare patients with TB with the rest of the population. As the statistical reviewer correctly noted in their comments (comment 42), such analysis might suffer from an inappropriate adjustment of confounders when describing the programmatic gaps.

TITLE
**7. Comment**: Please revise your title according to PLOS Medicine's style. Your title must be nondeclarative and not a question. It should begin with main concept if possible. "Effect of" should be used only if causality can be inferred, i.e., for an RCT. Please place the study design ("A randomized controlled trial," "A retrospective study," "A modelling study," etc.) in the subtitle (ie, after a colon).
**Response**: As the editor suggested, we modified the title of the manuscript with the updated title as follows: “Hepatitis C care cascade among patients with and without tuberculosis: Nationwide retrospective cohort study in the country of Georgia, 2015-2020”

ABSTRACT
**8. Comment:** Please structure your abstract using the PLOS Medicine headings (Background, Methods and Findings, Conclusions).
Please combine the Methods and Findings sections into one section, “Methods and findings”.
**Response**: We modified the structure of the abstract using the PLOS Medicine headings and combined the methods and findings into one section (Lines 54-79)

Abstract Background:
**9. Comment:** Line 122 of your main introduction details the objectives of your study slightly different to those presented in the abstract.  Please amend the abstract accordingly for consistency, clarity and to better inform the reader of the abstract.

**Response**: Thank you for noting the discrepancy. We updated the background section of the abstract to make it consistent with the introduction section (Lines 49-51).

Abstract Methods and Findings:
**10. Comment**: Please ensure that all numbers presented in the abstract are present and identical to numbers presented in the main manuscript text.

**Response**: We double-checked the abstract and main text and found a minor discrepancy in numbers, caused by omitting the number of people who started hepatitis C treatment before TB diagnosis. We updated the abstract to clarify that 9,065 patients are those without hepatitis C treatment before TB diagnosis, which aligns the numbers between the abstract and results section (lines 64-63).

**11. Comment**: Please elaborate on the details of the population studied and the setting, as you have done in the main methods section.

**Response**: We added the requested details in the abstract. (Lines 56-59)

**12. Comment**: Please quantify the main results for the outcomes measured with 95% CIs and p values as well as hazard ratios and the risk estimates that you describe in the main methods section.

**Response**: Per the editor’s request, we added more findings that include 95% CIs and p-values for the risk factor analysis and time-to-event analysis (lines 71-79). In response to one of the reviewer’s similar comments (Comment 81), we also include the additional table in the supplementary material for reviewers to show the p-values for the care cascade comparisons. However, as we described above, finding a causal association was not our purpose in the care cascade analysis, thus, we did not include that table in the main results or online supplement to avoid confusion in the reader.

**13. Comment**: Please ensure that you include the actual amounts and/or absolute risk(s) of relevant outcomes (including NNT or NNH where appropriate), not just relative risks or correlation coefficients. (example for absolute risks: PMID: 28399126).

**Response**: Since measures of absolute risk require a defined follow-up time (e.g., 2-year risk, 5-year risk) while our cohort consisted of patients with varying follow-up times, we did not calculate absolute risk in our study. Additionally, we believe that NNT and NNH measures are not relevant in the care cascade analysis. We will be more than happy to address any further comments regarding these additional measures if the editor would like to elaborate on which analyses should be expanded to include these measures.

**14. Comment**: Please include the important dependent variables that are adjusted for in the analyses.

**Response**: We added the details about the variables that were adjusted for in the analysis of risk factors for a given variable that we report in the abstract, MDR TB. (Line 75)

**15. Comment**: In the last sentence of the Abstract Methods and Findings section, please describe the main limitation(s) of the study's methodology.

**Response**: We added a sentence with the main limitation of the study, stating the following: “The main limitation of this study was that due to the reliance on existing electronic databases, we did not have all necessary variables to adjust for potential confounders in some of the analyses.” (Lines 77-79)

Abstract Conclusions:
**16. Comment**: Does the study have impact outside of Georgia?  If so, it would be helpful to include this

**Response**: We believe that findings from this study have implications for other countries, especially ones that currently implement hepatitis C elimination programs. We modified the conclusions section of the abstract with the following text: “Better integration of TB and hepatitis C care systems can potentially reduce LTFU and improve patient outcomes both in Georgia and other countries that are initiating or scaling up their nationwide hepatitis C control efforts and striving to provide personalized TB treatment.” (Lines 81-86)

AUTHOR SUMMARY
**17. Comment**: At this stage, we ask that you include a short, non-technical Author Summary of your research to make findings accessible to a wide audience that includes both scientists and non-scientists. The Author Summary should immediately follow the Abstract in your revised manuscript. This text is subject to editorial change and should be distinct from the scientific abstract. Please see our author guidelines for more information:
<https://journals.plos.org/plosmedicine/s/revising-your-manuscript#loc-author-summary>

**Response**: As requested by the editor, we added the author summary text in the manuscript file, below the abstract. (Lines 90-106)

METHODS and RESULTS

**18. Comment**: Did your study have a prospective protocol or analysis plan? Please state this (either way) early in the Methods section.
a) If a prospective analysis plan (from your funding proposal, IRB or other ethics committee submission, study protocol, or other planning document written before analyzing the data) was used in designing the study, please include the relevant prospectively written document with your revised manuscript as a Supporting Information file to be published alongside your study, and cite it in the Methods section. A legend for this file should be included at the end of your manuscript.
b) If no such document exists, please make sure that the Methods section transparently describes when analyses were planned, and when/why any data-driven changes to analyses took place. 
c) In either case, changes in the analysis-- including those made in response to peer review comments-- should be identified as such in the Methods section of the paper, with rationale.

**Response**: this study was conducted as part of a dissertation project. Before data analysis, a methodology and statistical analysis plan was written out in the dissertation proposal. We are submitting with the revised manuscript the portion of the dissertation proposal relevant to this manuscript in the file “data analysis plan”. We added a detailed description of all the changes that occurred after the proposal, stage when these changes occurred, and the rationale for those changes.

**19. Comment**: For all observational studies, we ask that in the manuscript text, you please indicate:
(1) the specific hypotheses you intended to test,
(2) the analytical methods by which you planned to test them,
(3) the analyses you actually performed, and
(4) when reported analyses differ from those that were planned, transparent explanations for differences that affect the reliability of the study's results. If a reported analysis was performed based on an interesting but unanticipated pattern in the data, please be clear that the analysis was data-driven.

**Response**: We made some additions to ensure that we request all of the required components:

(1) We added the prespecified hypothesis after the first objective in the last paragraph of the introduction. We did not have a prespecified hypothesis for the second objective. (lines 163-166).
(2) The analytical methods for each of the planned analysis is provided in the statistical analysis section.
(3) The analyses performed are also extensively described in the statistical analysis section.
(4) We are submitting with this revision the analytical plan developed before data analysis. In the annex of the analysis plan, weprovided a detailed list of all major changes from the planned analysis and described the reasons for those changes.

**20. Comment**: Where you report 95% CIs, please also report p-values and the statistical test used to determine them.  Suggest reporting as follows, for example, line 266: “…with TB (HR = 1.46, 95% CI: 1.39, 1.54)…” suggest reporting as “(HR: 1.46, 95% CI [1.39, 1.54], p< or =) please specify the significance level i.e. 0.05, 0.01.  Please amend throughout the main manuscript text and the abstract and the author summary.

**Response**: We added p-values throughout the manuscript in every result that reported a measure of association and confidence interval.

TABLES and FIGURES
**21. Comment**: We agree with the reviewer than inclusion of the DAG would be helpful.  Please see below and include as a supplementary file

**Response**: We agree with the inclusion of DAGs and submitting them in the supplementary material.

**22. Comment**: We ask all authors to provide a separate table showing the complete baseline characteristics of the study population, please include

**Response**: We added baseline characteristics of patients with TB and patients without TB in the supplemental material. Due to the difference in variables available for these two populations, we are including these results as three separate tables – persons diagnosed with TB, persons without TB who were screened for HCV antibodies, and persons without TB with positive HCV antibody results (Supplemental material, tables S1 S2 and S3).

**23. Comment**: Please ensure all figures/tables have appropriate captions describing their contents and defining any and all abbreviations including those used for statistical reporting including the supplementary files.

**Response**: We added all the necessary captions and abbreviations to figures in the main text and supplemental material.

**24. Comment**: Figure 1: please ensure all numerical values are identical to those presented in the manuscript text.

**Response**: We verified that all numerical values presented in the text and figure are the same.

**25. Comment**: Figure 2: please see statistical reviewer comments below, which we agree with.  It is unclear what all the numerical values on this figure refer to.  Please revise accordingly.  Please clearly define the meaning of the bars and whiskers in an appropriate caption.  Results should be quantified using p-values and 95% CIs.  Please apply the same revisions to the figures in the supplementary files which are presented in the same manner (figure S2)

**Response**: We added a footnote for the figure to clarify that the red lines represent the percent change between adjacent bars on the graph. As we responded above in comment 12, we did not conduct significance testing for the care cascade comparison. Per the editor’s request, we conducted the significance testing for the difference of proportions at each step of the care cascade and included the table in the supplemental material for reviewers (Table SR1). We will be happy to consider moving that table to online supporting information or in the main text if the editor could elaborate on the purpose of the significance testing for the given analysis.

**26. Comment**: Table 2: we agree with the reviewer that a label other than “other” would be preferable, if possible, when describing regions.  Where you report 95% CIs please also report p-values in a separate column and in the table caption please define the statistical test used to determine them.

**Response**: We changed the label of “Other” to “Rest of Georgia”. We also changed the variable name from “region” to “Location”, considering that the third category in that variable is penitentiary system which technically is not a region. We added two columns to tables 2 and 3 with p-values from unadjusted and adjusted analyses.

DISCUSSION
**27. Comment**: Line 308: “globally.[17]” please ensure in-text reference callouts are placed before punctuation in square parentheses: as follows “globally [17].”  Please check and amend throughout.

**Response**: We reviewed the reference callouts throughout the text and fixed the punctuation placement to ensure that all callouts were placed before punctuation.

**28. Comment**: Line 315: “[26, 28]” please remove spaced between citations as follows “[26,28]”

**Response**: We followed the guidelines for referencing provided on the web page of Plos Medicine, also mentioned by the editor in comment 29. As per the guideline, we used the reference management program Endnote and the style **Plos**. As we found, the default settings for that style automatically put space between two reference numbers even if we manually deleted the space. Please advise if we need to remove the endnote formatting and format the references manually or can keep the references with spaces (it seems like articles published Plos Medicine have space in between citation numbers).

REFERENCES
**29. Comment**: Please see our guidelines for referencing here: <https://journals.plos.org/plosmedicine/s/submission-guidelines#loc-references>
Journal name abbreviations should be those found in the National Center for Biotechnology Information (NCBI) databases.
In the bibliography please ensure that up to but no more than 6 authors are listed followed by et al, where more than 6 authors contribute to the study

**Response**: We used the reference management software Endnote and referencing style **Plos** to insert references and ensure that reference formats follow the Plos Guideline. We verified that no more than six authors are listed followed by et al, where more than six authors contribute to the study.

PARACHUTE RESEARCH POLICY
**30. Comment**: We note that you conducted research or obtained samples in a foreign country. Did you consider including a local author as first or last author? If not, we recommend that you consider doing so in line with ICMJE's authorship requirements (<https://www.icmje.org/recommendations/browse/roles-and-responsibilities/defining-the-role-of-authors-and-contributors.html>). PLOS has a parachute research policy which aims to promote collaboration and inclusivity in global health research. You are required to complete PLOS’ questionnaire on inclusivity in global research and submit it with your revised paper. The policy and questionnaire can be found at <https://journals.plos.org/plosone/s/best-practices-in-research-reporting>.

**Response**: We appreciate Plos for prioritizing the promotion of collaboration and inclusivity in global health research. This study involved local collaborators, such as researchers, clinicians, and statisticians from the country of Georgia, where the data was generated. Overall, 9 out of 15 authors of this paper, including the first author, are from the country of Georgia. We also completed the PLOS questionnaire on inclusivity, which is submitted with this revision.


Comments from the reviewers:

Reviewer #1: Thanks for the opportunity to review your manuscript. My role is as a statistical reviewer, so my review concentrates on the study design, data, and analysis that are presented. I have put general questions first, followed by queries relevant to a specific section of the manuscript (with a page/line reference).

This manuscript presents a cohort study of people utilising care for Hep C in the country of Georgia from 2015-2020, specifically comparing those also with TB. In this context (Georgia), there has been a program to eliminate Hep C from 2015, with free testing and treatment for citizens. There is evidence that Hep C is more commonly encountered in those with TB, but without formal referral pathways for TB patients. The manuscript describes the cascade of care for Hep C, and investigates personal factors for association with drop-out of the Hep C care system. The study uses data from a notifiable diseases database (TB), a screening registry (Hep C screening), a clinical database (Hep C testing and treatment), and the national death registry. Linkage between the databases was accomplished with deterministic linkage on a national ID. The drop-out was measured as time between a positive test to initiation of treatment, and analysed with a Fine-Grey model.

**31. Comment**: Was an analysis plan created for the study? Is this available to see as part of the review?

**Response**: As responded above to the academic editor (Comment 18), this study was conducted as part of a dissertation project. Before data analysis, the methodology and statistical analysis plan was written out in the dissertation proposal. We are submitting with the revised manuscript the portion of the dissertation proposal relevant to this manuscript. We added a detailed description of all the changes that occurred after the proposal, the stage when these changes occurred, and the rationale for those changes.

**32. Comment**: I would consider adding the study design (e.g. retrospective cohort study) to the title of the manuscript.

**Response**: We agree with the reviewer and updated the manuscript title. The updated title of the manuscript: “Hepatitis C care cascade among patients with and without tuberculosis: Nationwide retrospective cohort study in the country of Georgia, 2015-2020”. We also added in the methods section that the study used retrospective cohort design. (Line 174)

**33. Comment**: Are there treatment services for TB and Hep C outside of the national programs, e.g., care provided privately by physicians?

**Response**: Hepatitis C medication (DAAs) is not commercially available in the country besides the national program, thus it can be assumed that the Hep C treatment service outside the program is almost nonexistent. The situation is similar with TB - although some of the medication can be purchased privately, the diagnostic and treatment infrastructure in the country is bult only for the national program, and since all TB-related services are free of charge for the patient only in the national program, it can be assumed that the number of patients treated privately is negligible.
**34. Comment**: In the methods I would include explicitly how missing data was dealt with in the analysis - e.g. missing as indicator in the LTFU analyses. This makes the assumption that the missing level of a variable is not correlated with any of other of the variables in the analysis (MCAR). Is this a reasonable assumption to make with this data? Is it possible to use an imputation or IPW strategy to be able to make only a missing-at-random assumption?

**Response**: We conducted a complete case analysis in the LTFU analyses. The missingness does not exceed 5% in any of the variables that we used in the LTFU analyses, as shown in tables 2 and 3. We believe that MCAR is a reasonable assumption with these data, there is no apparent way that missingness could be associated with any variable in the analysis. We would greatly appreciate the statistical reviewer’s further comment on whether <5% is an acceptable level of missingness for complete case analysis and if not, which method should we pursue. We added the description of how the missing data was dealt in the methods section. (Lines 266-267)

**35. Comment**: P7, L114. Are the TB services offered to everyone, or limited to citizens (like the Hep services)?

**Response**: Services are offered to all residents of Georgia, not only citizens. We added that clarification in the text (line 154)

**36. Comment**: P7, L133. What does 'NTP' stand for?

**Response**: It stands for National Tuberculosis Program. The acronym is defined in line 153

**37. Comment**: P10, L191. Was mortality relatively common in the cohort? Is it possible to see the censoring reason (end of follow-up, mortality) by TB status to help assess if competing risks are likely to be an issue.

**Response**: Some information on mortality was already provided in the results section, but we added a few more sentences to describe the extent of mortality both in patients with TB and without TB. Among patients with TB and positive antibody test, 6% died without viremia testing. Among those who had positive viremia test, 8% died. As for patients without TB, 3% of antibody positive patients died without receiving confirmatory viremia testing and 4% of those with viremia died without receiving treatment. These additional data are provided in the revised manuscript on lines 314, 318, 324-326.

**38. Comment**: P10, L192. To clarify, was a standard cox model used to estimate HRs, rather than a subdistribution HR?

**Response**: The subdistribution hazards model was used for the analysis. We clarified that in the statistical analysis section, and the updated text now reads: “We used subdistribution hazards model to calculate hazards ratios (HR) and assess the differences between groups numerically. Death was treated as a competing risk”. (Lines 247-248)

**39. Comment**: P11, L207. It's good to see covariate selection based on a DAG - is it possible to include this in the supplementary material?

**Response**: We appreciate the reviewer requesting DAGs and included them in the supplementary material (Supplementary material figures S1-S4)

**40. Comment**: P11, L219. Is this missing ID likely to come from incomplete collection, or because those patients did not have a national ID (e.g. non-citizens)?

**Response**: It was likely due to combination of both; we do not have exact reasons for this missingness.

**41. Comment**: P14, L287. 'multivariable' is the preferred term for this analysis ('multivariate' is used with multiple outcomes or with mixture models)

**Response**: We agree with the reviewer that the term “multivariable” is preferred for this analysis. We made sure that the term was used correctly throughout the paper.

**42. Comment**: P18, L378. While this is a limitation to making a causal inference, I do think there being able to describe gaps between TB and non-TB patients as you have done is still very useful, and that if establishing a treatment gap then inappropriate adjustment of confounders (which effectively end up justifying a gap because someone is older, from a rural location etc.) is usually the problem.

**Response**: We agree with the reviewer that this limitation is true for the causal inference. Even without confounder adjustment, the gaps seen in this analysis are an important finding with practical implications. We added the following sentence in the given part of the discussion section: “However, even without exploring the causal association, the pronounced difference between patients with both TB and hepatitis C and those with only hepatitis C warrants the attention to reduce the gap in linkage to hepatitis C care and treatment among patients with TB.” (Lines 511-514)

**43. Comment**: P29, Table 2. Is Tbilisi the only major urban region in the country? Is there another label for the 'other' category that is more informative?

**Response**: Yes, Tbilisi is the capital and major urban are of the country. There are other, relatively smaller cities, and the country is divided into 10 regions besides Tbilisi, but due to very small cell counts in some of those regions, we decided to combine the rest of the regions into one category in the main analysis. We changed the name of the variable from “Region” to “Location” and renamed the category “other” to “Rest of Georgia”.

**44. Comment**: P36, Figure 2. What are the error bars that are separate from the bars on these figures? It might be better to include the estimate of number of patients as a dot with error bars placed directly on the dots. I would also consider reporting the cascade as a rate (%) rather than as remaining sample.

**Response**: Thanks for the suggestion. We created a new figure comparing the care cascade between patients with and without TB as a percentage at each step rather than as remaining sample. We are adding this to the supplementary material (figure S5). As for the error bars, the lines separating the adjacent bars represent the percent change between the two steps in the care cascade. We added this clarification in the figure captions.

**45. Comment**: P38, Figure 2. Is it possible to include a confidence interval for the cumulative incidence estimates?

**Response**: Unfortunately, the SAS procedure that we used for cumulative incidence graphs does not generate the confidence interval bands. In response to reviewer’s comment, we generated similar graphs with Kaplan-Meier approach, which we are submitting in the file “supplementary material for the reviewers”. Although not exactly inverse of the cumulative incidence curves, these KM graphs give a general idea of the width of the confidence intervals. We are happy to consider moving the graphs in the supporting information to be posted online, if the reviewer thinks this is relevant and interesting for the reader.


Reviewer #2: Thank you for the opportunity to review this paper. The authors have presented an important topic and have well described their results and conclusions. I have put the specific comments in the pdf version of the article. Specifically I would mention that I think the cascade should be in the main body of the article whereas the tables of regression do not need to be ( and can be in the supplement).
**Response**: We appreciate the reviewer’s valuable comments and addressed them in our revised manuscript. For the ease of review, we are pasting the reviewer comments below and providing the response on each of them.

**46. Comment**: The title is good but the initial explanation of the objectives does not reflect that; later in the article it is better reflected. Therefore I would ask the authors to check the text where they have mentioned what the objective of the article is and make sure that it corresponds to the title ( and the later described objectives.
Thanks.

**Response**: Thank you for raising this issue. We updated the objectives in the abstract (lines 49-53) to correspond to the objectives in the main text and the title.

**Abstract**

**47. Comment**: this does not correspond to the title (referring to the objective)

**Response**: We agree with this comment which was also raised by another reviewer. We updated the wording in the abstract to make it correspond to the objective in the main text and the manuscript title. (Lines 49-53)

**48. Comment**: better to say "amongst those who were tested" or "of which" . in terms of the cascade analysis then it should be clear what the denominator is

**Response**: We agree with the reviewer’s suggested language and added the words “of which” to clarify the denominator (line 63).

**49. Comment**: This is hard to follow in the abstract as you now give results on cure whereas until now, all was about LTFU.

**Response**: We understand that the wording might be confusing because the focus of the text switches from LTFU to cure, but we think that the given sentence is important to keep in the abstract since it represents the overall impact of the LTFU on the final outcome in the cascade – cure. To make it more tied to the previous sentences, we added the phrase “due to LTFU at various stages across the care cascade”. (Line 67)

**Introduction**

**50. Comment**: Line 75 - this is not relevant

**Response**: We agree with the reviewer and deleted the given sentence (Line 114-116)

**51. Comment**: Line 89 - this is not correct. Historically before DAAs treatment with interferon was not recommended when a patient had TB because of immunosupressive effects - nothing to do with drug drug interactions. DAAs are only recent history

**Response**: We appreciate the reviewer’s insight into correcting our statement. We expanded the sentence to clarify those reasons for not recommending parallel treatment. The updated sentence now reads: “Historically, patients receiving treatment for TB have not been recommended to receive hepatitis C treatment until they finish TB treatment due to immunosuppressive effect of interferon or due to drug-drug interactions of more recently introduced directly-acting antivirals”. (Lines130-131)

**52. Comment**: Line 120 - is this the case now or was it the case in 2018?

**Response**: This is still the case now, there is no formal referral system for the linkage of TB patients to hepatitis C care. We added the word “currently” to clarify this. (Line 160)

**53. Comment**: Line 122 - this is not the same as in the introduction

**Response**: We assume the reviewer refers to discrepancy between the abstract and the introduction in terms of how objectives were framed. We updated the background portion of the abstract to make the objectives consistent with the introduction. (Lines 49-53)

**Methods**

**54. Comment**: Line 140 - Any reason why you chose 2 different cut off dates?guess it is because the TB cohort is the start date of TB so you allow 9 months for the patient to have a HCV test? You should explain that

**Response**: The reviewer is right; we did not include patients with TB diagnosed in 2020 because they would have very limited follow-up time for HCV-related procedures. We added a sentence with this explanation in the “Study population” section. (Lines 49-53)

**55. Comment**: Line 145 - Since the whole article is on the cascade I would expect the cascade to be in the article itself. Then only details in the text

**Response**: We agree with the reviewer’s suggestion and moved the figure describing the care cascade in the main text. (Line 196)

**56. Comment**: Line 146 - can you name the tests used?

**Response**: We added a phrase to clarify that antibody tests can be either rapid or laboratory-based (line 191). It is challenging to provide a more detailed description of tests since the screening for hepatitis C in Georgia is conducted in multiple locations and within various settings and programs. Thus, there is a wide variety of manufacturers of tests used throughout the country.

**57. Comment**: What about the first step of how many people who should have been tested for HCV antibodies were tested? this is missing in the graph in the supplement. I would also put the graph in the article not in the supplement as it is the whole objective

**Response**: We agree with the reviewer’s suggestion and moved the supplemental figure to the main text. As for adding an extra step in the beginning, we want to clarify that the figure describes the programmatic steps that a patient has to go through in the hepatitis C care cascade. Each step represents an action within the program (e.g., testing, treatment initiation). Therefore, HCV screening through antibody testing is the first step in this process. A total number of people that should be tested does not fit in the cascade the way we present it. We clarified in the title of the figure that this figure represents the steps in the elimination program. (Line 196)

**58. Comment**: Line 153 - why dont you start with all TB patients and consider how many had a HCV test - this would be the beginning of the cascade ( although it is not the part of the cascade comparable with the non TB patients it is still important. Perhaps you mention this further down

**Response**: We agree with the reviewer about the importance of describing how many patients with TB had HCV test performed. In fact, we describe this extensively in the results section, in the hepatitis C care cascade subsection, thus, this information is provided in the text but is not included in the care cascade graphs for two reasons. (1) As the reviewer correctly noted, this step is not comparable with the non-TB population, whereas we wanted to cascade graphs to serve for the comparison. (2) since the number of patients with TB is much higher than the number of patients with positive HCV antibody results, adding bars with the total number of patients with TB and the total number of those who were screened distorts the size of the other bars so that the differences is not pronounced anymore. Since the main focus of our care cascade analysis is the LTFU after positive antibody test results and after positive viremia test, we want to make sure that those steps are visible. Just as an example, we provided the figure of the care cascade starting from the total number of patients with TB in the file “Supplementary materials for the reviewers”.

**59. Comment**: Line 153 - any time after TB diagnosis - ie no cut off - could be 2 years? make it clearer

**Response**: We included HCV antibody testing any time after TB diagnosis. Per the reviewer’s request, we clarified this by adding the words “any time” in the given sentence. (Lines 202-203)

**60. Comment**: Line 164 - to check later this is mentioned in the limitations - the biases of excluding those who died (could go both ways)

**Response**: The reviewer raises an important point. We would like to clarify that in the time-to-event analysis, we used statistical methods that take into account death as a competing event. We added that information in the methods section under statistical details. (Lines 246-248)

**61. Comment**: Line 187 - not clear how you handled the fact that DAA were not given during DS-TB treatment

**Response**: Originally, we included the graph where follow-up started at the time of viremia testing for everyone. In the revised version of the manuscript, we include the graph which starts from the TB treatment completion for those patients that had viremia-positive results during their TB treatment. The graphs are almost identical, but we agree with the reviewer that the fact that the TB patients were not eligible for the HCV treatment should be taken into account in this analysis. We also added this clarification in the methods section: “For patients with TB who had positive viremia test during their TB treatment, follow-up time started at the end of their TB treatment.” (Lines 243-245)

**Results**

**62. Comment**: Line 229 - are the characteristics of the 2 groups in a table somewhere? you mention different characteristics of the 2 groups in the text so I imagine there is a table where we can see all the characteristic of the 3 groups side by side?

I see later you don't have the same data for the HCV population but you should maybe mention it earlier?

**Response**: As the reviewer correctly noted, we do not have much information that could be compared between patients with and without TB, so adding an extra table to compare just age and sex would not offer a comprehensive comparison between the groups. We agree with the reviewer that this should be mentioned earlier, so we added the following sentences in the methods section: “We conducted descriptive statistics of patients with and without TB to describe available demographic and clinical characteristics (Supplemental tables S1, S2, S3). Due to a limited number of variables in HCV screening registry, we could not conduct extensive comparison of the two groups”. (Lines 236-239)

**63. Comment**: Line 233 - can this be put into the graph on the cascade? I understand that it is good to have the same graph for the non TB so you can't include this - but perhaps in the supplement you can put an additional graph that includes this?

**Response**: We responded to a similar comment extensively above (see comment 58). We created a figure which includes these additional numbers in the care cascade and providing it in the supplementary material for the reviewers. If reviewers and editor deem it necessary, we can include it in the supplemental material to be posted online.

**64. Comment**: Line 235 - mention the table here before describing the content

**Response**: We agree with the reviewer’s comment and added a mention of the table in the given line. (line 304)

**65. Comment**: Line 240 - do you mention also that the positivity rate went down over that period too?

**Response**: We added a sentence describing how the positivity rate decreased over time. The added sentence reads as follows: “In the same period, HCV antibody-positivity in this population decreased from 25% among people diagnosed with TB in 2015 to 14% among those diagnosed in 2019.” (Lines 308-310)

**66. Comment**: Line 274 - not clear if we are counting from the end of TB treatment since DAA cannot be given with rifampicin for the DS-TB patients?

**Response**: We appreciate the reviewer raising an important point. As we responded above in comment 61, we initially started counting everyone's follow-up time from the viremia testing. For this revision, we modified the methods to start the follow-up for patients with TB after their TB treatment completion. We updated the results section accordingly and replaced the old number with the new results. (Lines 365-368)

**67. Comment**: Line 279 - You dont do the same analysis for patients without TB - because you do not have this information?

**Response**: That is correct, especially among persons who were LTFU after HCV screening, we have very limited information. Furthermore, although we provide some level of analysis on patients without TB, that only serves the purpose of comparison, and our main focus is patients with TB. Therefore, analysis focused solely on patients without TB is beyond the scope of our study.

**Discussion**

**68. Comment**: Line 371 - reference 20 is not relevant here when mentioning the cohorts

**Response**: We agree with the reviewer that reference 20 was misplaced. We moved it to earlier in the sentence, where we mention that there is no documented drug-drug interaction between DAAs and second-line TB drugs. (Line 485)

**69. Comment**: Line 575 - these tables could go in the supplement

**Response**: Thank you for the suggestion. Since identifying the risk factors for LTFU among patients with TB was one of the two main objectives that we stated earlier in the manuscript, we prefer to keep these tables in the main text for now. However, if the editors request to reduce the number of tables and figures, we agree that these two tables are the main candidates to be moved to supplement.

**70. Comment**: Figure 2 - each graph needs a title

**Response**: We agree with the reviewer’s suggestion and modified the graphs. Instead of providing the description of each of the panel in the overall figure title, we added titles to each of the three graphs.

**Supplementary material**

**71. Comment on figure S1**: Rather see this in the article

**Response**: We agreed with the reviewer and moved the figure from supplemental material to the main text as figure 1 (Line 196)

**72. Comment on figure S2**: The title of the graph should be changed – it is among DS-TB patients seropositive HCV

**Response**: We added the words “HCV seropositive” in the title of the figure (currently figure S6). We want to clarify that the figure contains two panels, one for DS and one for DR TB, not only DS TB.

**73. Comment on figure S2**: Does the drop of 6% for DSTB and 9% for DR-TB mean that those patient died before testing? Is this the only reason that patients who were HCV antibody positive were not eligible for viremia testing?

**Response**: That is correct, the drop represents the proportion of patients dying without viremia testing. That is the only reason for exclusion for the rest of the care cascade.

**74. Comment on figure S2**: What about the number of patients eligible for HCV testing – i.e., the first column is missing – how many patients who should have been tested for HCV antibodies were tested?

**Response**: We addressed this question above in response to comment 58 about the similar graph in the main text.

**75. Comment on figure S2**: Can you add another graph in the supplement that also include that column for the TB patients?

**Response**: We are providing the graph as requested by the reviewer, i.e., including the column with the total number of TB patients. We believe it is not visually very informative so for now providing it as supplementary material for reviewers. We can include it in an online supplement in case the editor or reviewer request.

Reviewer #3: Thank you for this well-written and important article. I recommend acceptance with minor revisions.

Methods
**76. Comment**: - line 191. You mention death was treated as a competing risk. Did you employ competing risk regression? If so, please explain which method (e.g. Fine-Gray) you used and whether your Hazard Ratios are in fact subdistribition Hazard Ratios.

**Response**: We conducted subdistribution hazards model with Fine & Gray approach. Therefore, we updated the methods section the clarify the method used to generate hazards ratios. The updated text now reads: “We used subdistribution hazards model to calculate hazards ratios (HR) and assess the differences between groups numerically. Death was treated as a competing risk” (Lines 246-248)

**77. Comment**: - Risk factors for LTFU in Tb-patients. Could you also assess these factors in Non-TB patients and compare?

**Response**: The variables available in the HCV screening registry is unfortunately very limited. Additionally, risk factors for LTFU in the general population was not the focus of our study. There is another study underway that aims to improve the linkage to care and identify the main barriers to enrolling in the treatment, which will have better data to assess the risk factors for LTFU.

**78. Comment**: - line 207: you mention directed acyclic graph theory for a-priori covariate selection. Could you provide the graph (e.g. in supplementary material) so readers understand the thought process?

**Response**: Thank you for the suggestion, we are adding the DAGs to the supplementary material. (Figures S1-S4)

Results
**79. Comment**: - line 227: Do you have more information (other than age and gender) for those without TB co-infection?

**Response**: The variables available in the hepatitis C screening registry is limited. We have information on age and sex, date and place of their screening and setting where they were screened (e.g., inpatient setting, blood banks, etc.).

**80. Comment**: - line 244: how many died between testing for viremia and treatment initiation?

**Response**: Among patients with TB, out of 1025 viremia-positive persons, 80 (7.8%) died without receiving hepatitis C treatment. We added that number in the text (Lines 318-319)

**81. Comment**: - line 250 - : Could you provide statistical tests of significance between the respective proportions of those with and without TB?

**Response**: We appreciate the reviewer raising this issue. Initially we did not include the significance testing for comparing the care cascades for several reasons: First, the aim of that particular analysis was not to test any hypothesis or find a causal association between TB disease and loss to follow-up from hepatitis C care. For practical implications, it does not matter whether the difference between the two groups is statistically significant. Second, with such a large number of individuals in the analysis, especially in the non-TB group, even small differences that are irrelevant from a clinical or policy perspective can be statistically significant, and more focus should be on the magnitude of difference, not statistical significance. That being said, per the reviewer’s request, we conducted significance testing and included it as an additional table with p-values for comparing TB and non-TB patients at each of the care cascade step. We include the table in the supplementary material for the reviewers. If the reviewer or editors think that information is valuable for the article, it can be included in the supplementary material to be posted online.

**82. Comment**: - line 264 - : Could you provide statistical tests of significance between the respective median follow-up times between those with and without TB?

**Response:** We conducted Wilcoxon rank-sum test to compare the medians between two groups of patients, and this information is added to both methods section and in the results section as requested by the reviewer (Lines 251-252 and 347)

**83. Comment**: - Table 2 and 3: Why are no adjusted RR provided for gender?

**Response**: Thank you for this interesting comment. We want to clarify that our analysis included a biologic sex variable, not gender. Therefore, since no other factor can affect a person’s biologic sex, there are no confounders that need to adjust for. If any variables are associated with sex and outcome of interest (LTFU), they will be on a causal pathway from sex to outcome and thus should not be adjusted for.

Discussion
**84. Comment**: - Could you contrast with data from HCV cascaded of care (non-TB) from other countries?

**Response**: We added a paragraph describing the findings from other countries and how Georgian data fits within the literature. (Lines 463-470)

**85. Comment**: - Could you discuss the role of HIV and also Injecting drug use in the management of TB and HCV, how this could impact the cascade of care and how integration of care for HIV and injecting drug use with TB and HCV services should ideally by designed and how this is currently done in Georgia.

**Response**: Georgia has a low prevalence of HIV infection overall and among patients with TB, and by itself, HIV infection does not have a meaningful impact on the overall hepatitis C care cascade. However, hepatitis C treatment is well-integrated into HIV care because the service providers are usually located in the same facilities and in many cases are the same providers. As for injecting drug use, antibody testing is routinely integrated into harm reduction settings, while viremia testing and treatment are not. We added all this information as a separate paragraph of the discussion (Lines 492-502).


Reviewer #4: Dear authors,

Your paper is presenting important set of data which can help shape up policies to address the gaps. I have the following queries:


86. **Comment**: Prisoners are highly vulnerable groups for TB and Hep C. Please elaborate in the text whether the data from TB and Hep C Elimination programmes include individuals who are currently in prisons (the penitentiary sector), (not with the history of incarceration). If so which proportions were prisoners during the dx and tx initiation.

**Response**: Thank you for raising an important point. Patients diagnosed in the penitentiary system are also included in the National TB program database and thus are included in our analyses. As for the hepatitis C diagnostics and treatment for current prisoners, the data from the penitentiary system is not linked to the rest of the nationwide data, so our non-TB data does not include current prisoners. We added this information in the “Sources of data” subsection of the methods section. (Lines 223 and 230)

87. **Comment**: Higher proportions of LTFU and delay in start of treatment after viremia testing among people with TB are important findings. What could be the cofounding factors (e.g. socioeconomic determinants, homeless, alcohol/substance use) and how they are ruled out.  Any statistical analysis to that end? If not this could be another limitation of the study. To your view what is the explanation of difference in male and female? how about MSMs?

**Response**: We agree with the reviewer about the importance of potential confounders if we want to explore the causal association between TB and LTFU from hepatitis C care. Unfortunately, due to the limited number of variables in the programmatic datasets and the difference in variables between TB and hepatitis C data sources, we could not adjust for the potential confounders mentioned by the reviewer (e.g., homelessness, substance abuse). We describe this as a limitation in the discussion section (lines 508-514). We would like to add that as noted by the statistical reviewer (reviewer 1) in comment 42 that, we fully agree, confounder adjustment is only relevant if we want to estimate causal associations, while describing the programmatic gaps between TB and non-TB patients, which was the main purpose of this analysis, does not require confounder adjustment.

88. **Comment**: Further clarify in the text which percentage of individuals on the data base had DR-TB

**Response**: We added in the results section that 11% of patients with TB had DR TB. (Line 290)

89. **Comment**: Which percentage of triple infection Hep C, HIV and TB exists in your setting? if this data is not available please mention it.

**Response**: We could not identify any previous evidence in the literature regarding the prevalence of triple infection in Georgia. In our sample, we found that of 1025 HCV viremia-positive individuals, 51 had positive HIV test results, corresponding to 5% out of those with TB/HCV coinfection and 0.6% out of all 9065 patients with TB who was also tested for HCV infection.

90. **Comment**: The below paper in Lancet Infectious Diseases had highlighted the need for integrated services. The same paper mentions Georgia as a good practice. Which elements of the mentioned practice has been working well and which are those need improvements or changes.

Dara, M., Ehsani, S., Mozalevskis, A., Vovc, E., Simões, D., Avellon Calvo, A., Casabona I Barbarà, J., Chokoshvili, O., Felker, I., Hoffner, S., Kalmambetova, G., Noroc, E., Shubladze, N., Skrahina, A., Tahirli, R., Tsertsvadze, T., & Drobniewski, F. (2020). Tuberculosis, HIV, and viral hepatitis diagnostics in eastern Europe and central Asia: high time for integrated and people-centred services. The Lancet. Infectious diseases, 20(2), e47-e53. <https://doi.org/10.1016/S1473-3099(19)30524-9>

**Response**: Thank you for suggesting this excellent and very relevant paper. The integration at the screening stage is quite widespread in Georgia and is indeed an example of good practice. Programs are not integrated the same way in the treatment stage. We added a paragraph in the discussion section with this information which references this paper on the integration of services. (Lines 492-502)
