# Supplementary material for: Hepatitis C care cascade among patients with and without tuberculosis: Nationwide observational cohort study in the country of Georgia, 2015–2020
Source: PLoS Med. 2023 May 4;20(5):e1004121. doi: 10.1371/journal.pmed.1004121 (PMC10194957; doi:10.1371/journal.pmed.1004121)
Supplement: Attachment — Submitted filename: Response to reviewers.docx [file pmed.1004121.s017.docx]

Authors would like to thank editors and reviewers for taking extra time reviewing our revised manuscript and providing additional feedback. We addressed all of the comments in the manuscript and provided the point-by-point response to each comment below. Line numbers references in our responses correspond to the marked-up file of the manuscript.

TITLE
**Comment 1**: Thank you for revising your title suggest “Hepatitis C care cascade among patients with and without tuberculosis between 2015 and 2020: A nationwide observational cohort study from the country of Georgia”

ABSTRACT

**Comment 2**: Response 13: “…NNT and NNH measures are not relevant in the care cascade analysis.”  We agree with you. This information was transposed from a checklist incorrectly. Please accept our apologies.

**Comment 3**: Line 50: please add “(LTFU)” to define the abbreviation used latterly.

**Response**: Thank you for noticing this; we added the abbreviation on line 50 where we first mention the term “loss to follow-up” (line 50).

**Comment 4**: Line 53: “…adults…” please clearly state the total number of participants included in your study here.

**Response**: We agree with the suggestion and added the total numbers of the study population in the abstract. The updated sentence reads as follows: The study population included 11,985 adults (aged ≥18 years) diagnosed with active TB from January 1, 2015, through December 31, 2019, and 1,849,820 adults tested for HCV antibodies between January 1, 2015, and September 30, 2020, who were not diagnosed with TB during that time (lines 53-55)

**Comment 5:** Line 60-66: “…20%...” of how many? Please clearly define numerators and denominators used to derive percentages to improve clarity for the reader.

**Response**: We added the numerators and denominators for percentages (lines 60-61).

**Comment 6:** Line 71: please define “MDR” here at first use

**Response**: We defined the acronym MDR at the first mention abstract (line 72).

**Comment 7:** Line 74: suggest “…we were unable to account for the impact of all confounding factors, such as…” or something similar

**Response**: Thank you for the suggestion. We modified the sentence that now reads as follows: “The main limitation of this study was that due to the reliance on existing electronic databases, we were unable to account for the impact of all confounding factors in some of the analyses” (lines 74-75). We did not go into details by naming the specific confounders because different analyses (e.g., time-to-event analysis comparing TB and non-TB population, LTFU analysis among patients with TB) have different sets of unknown confounders, and abstract word count does not allow us to add any more text (current word count is 498/500).

AUTHOR SUMMARY
**Comment 8**: Thank you for including an author summary. Please structure your summary according to PLOS Medicine’s style. Please see our author guidance here <https://journals.plos.org/plosmedicine/s/revising-your-manuscript#loc-author-summary>

We encourage you to see our website here <https://journals.plos.org/plosmedicine/>  for examples in published research articles.

The author summary should include ideally 2-3 (but no more than 4) single sentence bullet points for each of the following questions. Bullet points should be objective, brief, succinct, specific, accurate, and avoid technical language.

Why Was This Study Done?
Authors should reflect on what was known about the topic before the research was published and why the research was needed.

What Did the Researchers Do and Find?
Authors should briefly describe the study design that was used and the study’s major findings. Do include the headline numbers from the study, such as the sample size and key findings.   

What Do These Findings Mean?
Authors should reflect on the new knowledge generated by the research and the implications for practice, research, policy, or public health. Authors should also consider how the interpretation of the study’s findings may be affected by the study limitations.

**Response**: Thank you for providing detailed guidance on the author summary. We updated the summary text according to the instructions (lines 84-111)

TABLES
**Comment 9**: Thank you for adding the tables detailing the baseline characteristics of the study population.  Please move these to the main manuscript.

**Response**: Thank you for the suggestion. We added the supplemental tables about baseline characteristics to the main text as tables 1, 2, and 3.

SUPPLEMENTARY MATERIAL FOR REVIEWERS
**Comment 10**: Thank you for including this information.  To help facilitate the transparency of data reporting, please include these files as supporting information to be published with the manuscript. Please ensure that files are affiliated to an appropriate caption that clearly describes their contents, including abbreviations. Please report p as <0.001 and where higher as p=0.002, figure SR3 for example

**Response**: Thank you for the suggestion. We moved all the tables and figures from “supplementary material for reviewers” to supplemental materials that can be published online and added captions and a list of abbreviations. We updated the p-values to include three decimal points. The only exception is p-values on Kaplan-Meier curves (supplemental figures S8 and S9) generated in SAS, where we were unable to adjust the decimal points of the p-value.

FIGURES
**Comment 11:** To make your figures, including those in the supporting files, more accessible to those with colour blindness please consider avoiding the use of red and/or green.

**Response**: We updated the graphs to limit the use of red and green.

DISCUSSION
**Comment 12:** Line 387: suggest “…with TB between 2015 and 2019...”

**Response:** We agree with the suggested edit and updated the text accordingly (line 403)

REFERENCES
**Comment 13:** PLOS Medicine does request that for in-text reference callouts there are no spaces between citations. Please amend (publication without is a formatting error, perhaps originating in the citation manager, which we will look into).

Response: Thank you for clarification. We updated the in-text reference callouts to remove spaces between numbers.

SOCIAL MEDIA
**Comment 14**: To help us extend the reach of your research, please provide any Twitter handle(s) that would be appropriate to tag, including your own, your coauthors’, your institution, funder, or lab. Please detail any handles you wish to be included when we tweet this paper, in the manuscript submission form when you re-submit the manuscript.

**Response**: We appreciate the PLOS Medicine helping to extend the reach of the research. With submission, we include the twitter handles of coauthors, funders, and institutions.

COMMENTS FROM THE ACADEMIC EDITOR
I agree with decision for minor revision.
**Comment 15**: My only other comment is to have clarity on changes in downstream processes over time. They describe increases in patients completed Ab testing and decreases in proportion who tested positive. Of those who tested positive, did f/u viral load testing change over time? Of those with confirmed HCV infection, did treatment linkage and initiation change over time?

**Response**: Thank you for this comment. The information about the downstream processes is spread throughout the manuscript so we agree with the editor that it would be helpful to have an overall statement summarizing the time trends in terms of testing and treatment. In the first paragraph of the discussion, we added the following text: “We also found that proportion of patients with TB who get HCV antibody testing and subsequent viremia testing increased in recent years, while proportion of those with viremia who receive treatment decreased” (lines 408-410). More specific details about the temporal trends in viremia testing are provided in the results section (lines 386-388), table 5, and one of the paragraph discussion section (lines 431-438). More specific details about the temporal trends in treatment initiation are provided in the results section (lines 393-395), table 6, and one of the paragraph discussion section (lines 454-463).


Comments from Reviewers:

Reviewer #1: Thanks for the revised manuscript and responses to my review. The revision covers most of the original queries from the first review.

I agree with the authors that NNH/NNT would not be appropriate applied to a cascade of care study. Also, I support the comment about adjustment for the biological sex variable (i.e. should not be adjusted for). I think the DAGs are a good inclusion and explain the choice of regression models.

**Comment 16**: It has been common in the past to rely on a specific threshold (e.g. 5%) for using complete-case analysis or missing as indicator. The issue with this is that the proportion of missing data does not fully capture how much information is lost, it's possible to lose a fairly small amount of missing information that is critical to getting an unbiased effect estimate (<https://doi.org/10.1016/j.jclinepi.2019.02.016>). For this analysis, there are several 'principled approaches' (<https://doi.org/10.1093/aje/kwx348>) that could be suitable. For predictors of LTFU in Hep C, and LTFU in HSV care, I would suggest multiple imputation would be reasonably easy to implement (with an MI model using the same covariates as the substantive analysis), with different types of variables (continuous and categorical) MICE would be a good choice. Most software packages now have the capability (e.g. proc MI in SAS, MICE package in R, mi procedures in Stata) and are straightforward to use.

**Response**: We appreciate the reviewer providing detailed suggestion on the strategy to use for the missing data. We agree with the use of multiple imputation and updated the multivariable analysis of LTFU before viremia testing and before treatment initiation. We conducted MI with fully conditional specification using the same covariates (lines 269-271). as in the substantive analysis and updated the results in tables 5 and 6 accordingly. The findings changed slightly for the models with employment, newly diagnosed TB, and treatment outcome. Models with MDR TB as exposure is unaffected since none of the variables in the model had any missingness.

**Comment 17**: P12, L257. What software was used in the analyses?

**Response**: We used SAS for all analyses and added this information in the manuscript text (line 272)

Reviewer #2: Thank you for the responses and corrections to the first review. I have no further comments on this version.


Reviewer #3: Thank you for the very thorough revision of the article. I recommend acceptance.


Reviewer #4: Thanks for your response to queries and having addressed suggestions.
